# PrediTALE: A novel model learned from quantitative data allows for new perspectives on TALE targeting

**DOI:** 10.1371/journal.pcbi.1007206

**Published:** 2019-07-11

**Authors:** Annett Erkes, Stefanie Mücke, Maik Reschke, Jens Boch, Jan Grau

**Affiliations:** 1 Institute of Computer Science, Martin Luther University Halle-Wittenberg, Halle, Germany; 2 Department of Plant Biotechnology, Leibniz Universität Hannover, Hannover, Germany; Ottawa University, CANADA

## Abstract

Plant-pathogenic *Xanthomonas* bacteria secrete transcription activator-like effectors (TALEs) into host cells, where they act as transcriptional activators on plant target genes to support bacterial virulence. TALEs have a unique modular DNA-binding domain composed of tandem repeats. Two amino acids within each tandem repeat, termed repeat-variable diresidues, bind to contiguous nucleotides on the DNA sequence and determine target specificity. In this paper, we propose a novel approach for TALE target prediction to identify potential virulence targets. Our approach accounts for recent findings concerning TALE targeting, including frame-shift binding by repeats of aberrant lengths, and the flexible strand orientation of target boxes relative to the transcription start of the downstream target gene. The computational model can account for dependencies between adjacent RVD positions. Model parameters are learned from the wealth of quantitative data that have been generated over the last years. We benchmark the novel approach, termed PrediTALE, using RNA-seq data after *Xanthomonas* infection in rice, and find an overall improvement of prediction performance compared with previous approaches. Using PrediTALE, we are able to predict several novel putative virulence targets. However, we also observe that no target genes are predicted by any prediction tool for several TALEs, which we term orphan TALEs for this reason. We postulate that one explanation for orphan TALEs are incomplete gene annotations and, hence, propose to replace promoterome-wide by genome-wide scans for target boxes. We demonstrate that known targets from promoterome-wide scans may be recovered by genome-wide scans, whereas the latter, combined with RNA-seq data, are able to detect putative targets independent of existing gene annotations.

## Author summary

Diseases caused by plant-pathogenic *Xanthomonas* bacteria are a serious threat for many important crop plants including rice. Efficiently protecting plants from these pathogens requires a deeper understanding of infection strategies. For many *Xanthomonas* strains, such infection strategies depend on a special class of effector proteins, termed transcription activator-like effectors (TALEs). TALEs may specifically activate genes of the host plant and, by this means, re-program the plant cell for the benefit of the pathogen. Target sequences and, consequently, target genes of a specific TALE may be predicted computationally from its amino acids. Here, we propose a novel approach for TALE target prediction that makes use of several insights into TALE biology but also of broad experimental data gained over the last years. We demonstrate that this approach yields a higher prediction accuracy than previous approaches. We further postulate that a strategy change from a restricted search only considering promoters of annotated genes to a broad genome-wide search is feasible and yields novel targets including previously neglected protein-coding genes but also non-coding RNAs of possibly regulatory function.

This is a *PLOS Computational Biology* Methods paper.

## Introduction

Many crop plants including rice can be infected by *Xanthomonas* bacteria causing disease in the affected plants, which results in substantial yield losses. Many strains of *Xanthomonas oryzae* pv. *oryzae* (*Xoo*) and *Xanthomonas oryzae* pv. *oryzicola* (*Xoc*) express a specific type of effector protein called transcription activator-like effectors (TALEs). TALE proteins function as transcription factors in infected host cells [1], and contain a nuclear localization signal, a DNA-binding domain, and an activation domain. The DNA-binding domain consists of tandem repeats that bind to the promoter of plant target genes. Each repeat consists of approximately 34 highly conserved amino acids (AAs), except for the amino acids at position 12 and 13, which are termed repeat variable diresdue (RVD) and are responsible for DNA specificity. The repeat domain forms right-handed superhelical structure, while the RVD is situated within a loop accessing the DNA [2, 3]. Each RVD binds to one nucleotide of the target box [4, 5], where amino acid 13 binds to the sense strand and amino acid 12 stabilizes the repeat structure. Hence, the specificity of each TALE is determined by its RVD sequence. In addition, most known target boxes are directly preceeded by a ‘T’, while ‘C’ and ‘A’ occur with decreasing frequencies, which is also referred to as “position 0” of the target box.

Some repeats deviate from the common length of 34 AAs and have, for this reason, been termed *aberrant* repeats. Aberrant repeats may loop out of the repeat array when a TALE binds to its DNA target box and by this means allow for increased flexibility, also binding to frame-shifted target boxes [6].

Different *Xoo* and *Xoc* strains express different repertoires of TALEs, where a single strain may host up to 27 TALEs [7–10].

Naturally occurring TALEs may activate susceptibility (S) genes that are responsible for bacterial growth, proliferation and disease development, but also disease resistance (R) genes [1].

The names of TALEs and TALE classes are based on the nomenclature introduced by the tool AnnoTALE [11]. TALEs are clustered according to the similarity of their RVD sequence and divided into classes.

Target boxes upstream of all known major virulence targets are located in forward orientation relative to the transcription start site (TSS). Recently, target boxes of TALEs have been reported to be also functional in reverse orientation relative to the transcription start site (TSS) of their target gene [12, 13]. However, reverse binding seems to be rather an exception than a general rule [13]. Accurate predictions of target boxes of TALEs are important for studying naturally occurring TALEs and determining their virulence targets, but also for the identification of target and off-target sequences of artificially designed TALEs. Over the last years, several tools have been designed for the *in-silico* prediction of TALE target boxes based on the RVD sequence of a given TALE and, subsequently, for the identification of target genes.

The TALE-NT suite includes “Target Finder”, a tool for predicting target boxes of TALEs based on their RVD sequence. It is available as online or command line application (http://tale-nt.cac.cornell.edu/) [14, 15]. In Target Finder, predictions are based on a position weight matrix calculated from frequencies of naturally occurring RVD-nucleotide associations. The user can choose whether the target box should start with nucleotide T or C.

Talvez is another prediction tool that uses PWMs to model RVD-nucleotide interactions [16]. It differs from Target Finder in deriving specificities of rare RVDs from those of common RVDs with the same 13th amino acid. Target sequences may only begin with nucleotide T or C, with a lower score assigned in the case of cytosine. In addition, Talvez may explicitly model that mismatches are tolerated to a larger degree if these are located near the C terminus [17]. Users of Talvez can choose between web-based and command line applications.

TALgetter [18] uses a local mixture model to predict TAL target sequences. The specificities were learned from 267 pairs of TALEs and target sites with qualitative information whether the pair is functional or not. According to Streubel *et al*. [19], the efficiencies of different RVDs are non-identical. The TALgetter model adapts a similar concept using an importance term, which is learned independently from the specificity of each RVD. TALgetter is implemented within the Java framework Jstacs [20], and is available as online and command line program.

In the web tool SIFTED [21], specificity data from a large-scale study using protein-binding microarrays (PBMs) were used for training model parameters. For this purpose, 21 TALEs constructed exclusively from the most common four RVDs (NI, HD, NN, NG) were designed and their binding specificity measured on ≈ 5,000-20,000 DNA sequences per protein using PBMs. However, we will not consider SIFTED in the remainder of this manuscripts, as the SIFTED web server is currently unavailable and the limited set of RVDs included into SIFTED does not cover the entire spectrum of those occurring in natural TALEs.

Predictions of all of these approaches still comprise a substantial number of false positive predictions, whereas some of the known target genes cannot be detected by these approaches. During the last years, several quantitative studies of TALE binding and transcriptional activation have been published. The studies included quantitative analyses of target gene activation by TALEs spanning naturally occurring RVDs [19, 22], specificities at position 0 of target boxes [23], complete exploration of all possible combinations of amino acids at RVD positions [24, 25], and systematic analyses of those RVDs frequently used in designer TALEs [21].

In this paper, we aim at developing a novel approach for modelling TALE target specificities based on these quantitative data. This approach, called PrediTALE, explicitly captures putative dependencies between adjacent RVDs, dependencies between the first RVD and position 0 of the target box, and also includes positional effects of mismatch tolerance. In contrast to previous approaches, model parameters are adapted by minimizing the difference between prediction scores and quantitative measurements for pairs of TALEs and target boxes. Like previous approaches, PrediTALE also predicts target boxes in reverse strand orientation relative to the TSS, but applies a small penalty term in this case, following the assumption that functional reverse target boxes are rather rare *in planta*. PrediTALE is the first approach to account for aberrant repeats when predicting TALE targets.

## Materials and methods

### Training data

Pairs of TALEs and putative target boxes were collected from systematic, quantitative experiments reported in [19, 22–25]. Data were further processed as detailed in S1 Text. Data were grouped by TALE, and the global weight was computed as the maximum assay value for the current TALE divided by the maximum assay value reported for all TALEs with the same 13th AA at any position in the current assay. Target values were computed as the assay value of the current pair of TALE and target box divided by maximum assay value over all tested target boxes for the current TALE.

While the normalization of target values has a mostly technical background as it simplified the selection of initial values during numerical optimization of our model (see below), the definition of global weights influences the optimization result. The choice of global weights has been motivated by the observation that some TALE architectures (e.g., those with long successions of identical RVDs, or 12th AAs not occurring in nature) show a generally lower activity than others, which also affects the influence of measurement noise and, hence, the reliability of assay values. With the choice of global weights proposed here, the influence of such TALEs on the final optimization result is reduced, while such TALEs do not need to be completely removed from the training set.

As detailed in S1 Text, PBM experiments from [21] were filtered for apparent data quality, normalized log-intensities were used as target values, and global weights were defined uniformly for all putative target boxes from a common PBM experiment.

### Bacterial growth conditions

*Xanthomonas oryzae* pv. *oryzae* (*Xoo*) strains PXO83, PXO142 and ICMP 3125^T^ were cultivated in PSA medium at 28°C.

### Plant growth conditions & inoculation

*Oryza sativa* ssp. *japonica* cv. Nipponbare was grown under glasshouse conditions at 28°C (day) and 25°C (night) at 70% relative humidity (RH). Leaves of 4-week-old plants were infiltrated with a needleless syringe and a bacterial suspension with an OD600 of 0.5 in 10 mM MgCl2 as previously described [26].

### RNA-seq data

Rice cultivar Nipponbare leaves were inoculated with *Xoo* strains PXO83, PXO142, ICMP 3125^T^, or MgCl2 as mock control in five spots in an area of approx. 5 cm using a needleless syringe. Two leaves of three rice plants each were inoculated for each strain and control, respectively. 24h later, samples were taken, frozen in liquid nitrogen, and RNA prepared. Three replicates of this experiment were done on separate days and subjected to RNAseq analysis, separately. Stranded libraries were sequenced on an Illumina HiSeq 2500 instrument (Eurofins Genomics) as 100 bp single-end reads

RNA-seq data 48h after inoculation with different *Xoc* strains (BLS256, BLS279, CFBP2286, B8-12, L8, RS105, BXOR1, CFBP7331, CFBP7341, CFBP7342), and mock controls [9] were downloaded from Gene Expression Omnibus available under accession number GSE67588.

RNA-seq data were adapter clipped using cutadapt (v1.15) [27] and quality trimmed using trimmomatic (v0.33) [28] with parameters “SLIDINGWINDOW:4:28 MINLEN:50”. Transcript abundances were computed by kallisto [29] using parameters “–single -b 10 -l 200 -s 40” and the cDNA sequences available from http://rice.plantbiology.msu.edu/pub/data/Eukaryotic_Projects/o_sativa/annotation_dbs/pseudomolecules/version_7.0/all.dir/all.cdna. Differentially expressed genes relative to the respective control samples were determined by the R-package sleuth [30].

For the *Xoo* strains and the respective mock control, replicates have been paired during library preparation and sequencing. Hence, the replicate was considered as an additional factor when computing p-values of differential expression for the *Xoo* samples but not for the *Xoc* samples. Differential expression was aggregated on the level of genes using the parameter target_mapping of the sleuth function sleuth_prep(), and b-value, p-value, and Benjamini–Hochberg-corrected q-value were recorded. The b-value reported by sleuth when applying a Wald test is actually a biased estimator of the log-fold change. However, as this is a more commonly understood term, we refer to the b-value as “log-fold change” in the remainder of this manuscript. Gene abundances, and sleuth outputs with regard to differential expression are provided as S1 and S2 Tables, respectively. RNA-seq reads were also mapped to the rice genome (MSU7) to obtain detailed information about transcript coverage. To this end, adapter clipped and quality trimmed reads were mapped using TopHat2 v2.1.0 [31], and the resulting BAM output files were processed in further analyes described below.

### Model

Let ***r*** = *r*_1_*r*_2_…*r*_*L*_ denote the RVD sequence of length *L* of a TALE, where *r_ℓ_* ∈ {*AA*, …, *YY*, A*, …, Y*} denotes a single RVD, and *r*_*ℓ*,12_ and *r*_*ℓ*,13_ denote the 12th and 13th AA of that RVD, respectively. Let ***x*** = *x*_0_*x*_1_…*x*_*L*_ denote a putative target box of length *L* + 1 of that TALE, where *x_ℓ_* ∈ {*A*, *C*, *G*, *T*} and *x*_0_ denotes the nucleotide bound by the zero-th, cryptic repeat.

The general idea of the model proposed here is to model the total binding score of a putative target box ***x*** given the RVD sequence ***r*** of a TALE as a sum of contributions of i) binding to the zero-th repeat, ii) binding to the first RVD, and iii) binding to the remaining RVDs, where the latter two terms may be weighted by an additional, position-dependent but sequence-independent term.

$$\begin{array}{ccc} s(x|r,\theta ) & = & m_{0}\left(x_{0}|r_{1},\theta_{0}\right)+m_{1}\left(x_{1}|r_{1},\theta_{1},\theta_{m}\right)\cdot p\left(1|\theta_{p}\right)+\\ & & \;\sum_{\ell =2}^{L}m\left(x_{\ell }|r_{\ell -1},r_{\ell },\theta_{m}\right)\cdot p\left(\ell |\theta_{p}\right) \end{array}$$ (1)

Here, ***θ*** = (***θ***_0_, ***θ***_1_, ***θ***_*m*_, ***θ***_*p*_) denote the sets of real-valued parameters of the term for binding to the zero-th, first, and remaining repeats, and the position-dependent term, respectively.

The term *m*_0_(*x*_0_|*r*_1_, ***θ***_0_) for binding to the zero-th repeat may depend on the first RVD on the TALE, since dependencies between zero-th and first repeat have been observed before [23]. However, our knowledge about such dependencies is limited to the data presently available and, hence, we limit the RVDs for which a dependency is considered to a set $R_{0}$. Our data regarding systematic, quantitative analyses of the base preference of the zero-th repeat is limited in general, although it is widely assumed that position 0 in target boxes of natural TALEs is preferentially *T* and less frequently *C*. We include this prior knowledge into *a-priori* parameters $\pi_{x_{0}}$.

$$\begin{array}{ccc} m_{0}\left(x_{0}|r_{1},\theta_{0}\right) & = & \pi_{x_{0}}+\theta_{0,x_{0}}+\delta \left(r_{1}\in R_{0}\right)\cdot \theta_{0,x_{0}|r_{1}} \end{array}$$ (2)

In this paper, we set $R_{0}=\left\{HD,NN,NG,NI,NS\right\}$ and *π*_*T*_ = log(0.6), *π*_*C*_ = log(0.3), *π*_*A*_ = *π*_*G*_ = log(0.05).

The term *m*_1_(*x*_1_|*r*_1_, ***θ***_1_, ***θ***_*m*_) for binding to the first repeat depends on the 13th AA *r*_1,13_ of the first RVD *r*_1_, but may be extended by additional terms that either model a general dependency on the complete first RVD (including the 12th AA), and/or a separate base preference for a given 13th AA at the first position. Again, this modularity allows us to adapt the model to the resolution of data available, since a substantial part of RVDs is only covered by the systematic but limited data reported in [24, 25].

$$\begin{array}{ccc} m_{1}\left(x_{1}|r_{1},\theta_{1},\theta_{m}\right) & = & \theta_{m,x_{1}|r_{1,13}}+\delta \left(r_{1}\in R_{1}\right)\cdot \theta_{m,x_{1}|r_{1}}+\\ & & \delta \left(r_{1,13}\in R_{2}\right)\cdot \theta_{1,x_{1}|r_{1,13}} \end{array}$$ (3)

In this paper, we set $R_{1}=\left\{HD,NN,NG,HG,NI,NK\right\}$ and $R_{2}=\left\{D,N,G,I\right\}$.

The term *m*(*x_ℓ_*|*r*_ℓ−1_, *r_ℓ_*, ***θ***_*m*_) for binding to the remaining repeats again depends on the 13th AA *r*_*ℓ*,13_ of the current RVD *r_ℓ_*, but may be extened by additional terms that either model a dependency on the complete RVD (with parameters shared with the correponding term used for the first RVD), and/or the complete RVD *r_ℓ_* at the current repeat and the 12th AA *r*_*ℓ*−1,12_ at the previous repeat:

$$\begin{array}{ccc} m(x_{\ell }|r_{\ell -1},r_{\ell },\theta_{m}) & = & \theta_{m,x_{\ell }|r_{\ell ,13}}+\delta \left(r_{\ell }\in R_{1}\right)\cdot \theta_{m,x_{\ell }|r_{\ell }}+\\ & & \delta \left(r_{\ell },r_{\ell -1}\in R_{3}\right)\cdot \theta_{m,x_{\ell }|r_{\ell },r_{\ell -1,12}} \end{array}$$ (4)

In this paper, we set $R_{3}=\left\{HD,NN,NG,NI\right\}$.

Finally, we define the position-dependent term as a mixture of two logistic functions and a constant term, where the logistic functions depend on the relative distance of *ℓ* from the start and end of the putative target box, respectively:

$$\begin{array}{ccc} p(\ell |\theta_{p}) & = & \frac{e^{\theta_{p,1}}}{\sum_{j=1}^{3}e^{\theta_{p,j}}}\frac{1}{1+e^{-\theta_{p,a,1}\left(\frac{\ell }{L}+\theta_{p,b,1}\right)}}+\\ & & \;\frac{e^{\theta_{p,2}}}{\sum_{j=1}^{3}e^{\theta_{p,j}}}\frac{1}{1+e^{-\theta_{p,a,2}\left(\frac{L-\ell }{L}+\theta_{p,b,2}\right)}}+\frac{e^{\theta_{p,3}}}{\sum_{j=1}^{3}e^{\theta_{p,j}}} \end{array}$$ (5)

The parameters *θ*_*p*,*a*,1_ and *θ*_*p*,*a*,2_ denote the slopes, and *θ*_*p*,*b*,1_ and *θ*_*p*,*b*,2_ denote the location parameters of the logistic functions.

The implementation of this model is available from the Jstacs github repository (cf. section “Availability”) in package projects.tals.linear.

### Learning parameters

The training data $D=(t_{1},\ldots ,t_{N})$ comprise tuples *t*_*i*_ = (***r***_*i*_, ***x***_*i*_, *v*_*i*_, *w*_*i*_, *g*_*i*_) of TALE RVD sequence ***r***_*i*_, target box ***x***_*i*_, target value *v*_*i*_, global weight *w*_*i*_ and group *g*_*i*_ (cf. sections “Data” and “Model”). Given the current parameter values ***θ***, we may further compute for each pair of TALE and target box, the corresponding model score *s*_*i*_ = *s*(***x***|***r***_*i*_, ***θ***_*i*_). The goal of the learning process is to adapt the parameter values ***θ*** such that the differences between computed scores *s*_*i*_ and target values *v*_*i*_ becomes minimal. However, despite the normalization of target values described in section “Data”, target values from different experimental setups (represented by the groups *g*_*i*_) may live on different scales. Hence, we allow the learning process to linearly transform the computed scores *s*_*i*_ before comparing them to the target values. The total error between target value and prediction score is defined as

$$\begin{array}{ccc} E(\theta ;D,\beta ) & ≔ & \sum_{i=1}^{N}w_{i}\cdot {\left(f\left(s\left(x_{i}|r_{i},\theta \right)|g_{i},\beta \right)-v_{i}\right)}^{2} \end{array}$$ (6)

where

$$\begin{array}{ccc} f(s_{i}|g_{i},\beta ) & = & \exp\left(\beta_{a,g_{i}}\right)\cdot s_{i}+\beta_{b,g_{i}}, \end{array}$$ (7)

***β*** = (*β*_*a*,1_, *β*_*b*,1_, …, *β*_*a*,*G*_, *β*_*b*,*G*_), $\beta_{a,g_{i}}$ and $\beta_{b,g_{i}}$ are group-specific scale and shift parameters, respectively, and *G* is the total number of groups in the data set D.

In addition, we use an *L*_2_ regularization term on the model parameters ***θ*** to avoid overfitting and explosion of parameter values:

$$\begin{array}{ccc} L_{2}\left(\theta \right) & ≔ & \lambda \cdot {\left||\theta |\right|}_{2} \end{array}$$ (8)

where the regularization parameter λ is set to 0.1 in this paper.

The number of model parameters for the different terms varies greatly, depending on the number of conditions (e.g., 12th AA of previous RVD, separate parameters for individual RVDs). This regularization also has the effect that more complex dependency parameters assume values considerably different from 0 only if the modeled specificity cannot be captured by the less complex sets of parameters.

The final objective function is then to minimize sum of the error term E(θ;D,β) and the regularization term *L*_2_(***θ***) with respect to the parameter values:

$$\begin{array}{ccc} \left(\theta^{*},\beta^{*}\right) & = & \underset{\left(\theta ,\beta \right)}{\text{argmax}}\;E\left(\theta ;D,\beta \right)+L_{2}\left(\theta \right) \end{array}$$ (9)

This objective function is implemented in class MSDFunction in package projects.tals.linear. Parameter optimization is performed by a gradient-based quasi-Newton method as implemented in class de.jstacs.algorithms.optimization.Optimizer of the Jstacs library [20]. As the objective function is not convex, we start the optimization from 50 independent, random initializations and finally choose the set of locally optimized parameters that achieves the minimum value of the objective function.

The final parameters θ* of the trained model may then be used to determine prediction scores of previously unseen pairs of TALEs and putative target boxes, whereas the value of β* is discarded after optimization.

### Prediction of TALE target boxes

For predicting putative TALE target boxes for a given TALE with RVD sequence ***r*** of length *L*, we follow a sliding window approach scanning input sequences ***x***_1_, …, ***x***_*N*_. Input sequences could, for instance, be promoter sequences of annotated genes but also complete chromosomes. Each sub-sequence ***x***_*i*,*ℓ*_, …, ***x***_*i*,*ℓ*+*L*_ then serves as input of the model to compute the corresponding score *s*(***x***_*i*,*ℓ*_, …, ***x***_*i*,*ℓ*+*L*_|***r***, θ*). To allow for a rough comparison of scores, even between TALEs of different lengths, we normalize this score to the length of the input sequence, i.e., we compute a normalized score as *s*′(***x***_*i*,*ℓ*_, …, ***x***_*i*,*ℓ*+*L*_|***r***, θ*) ≔ *s*(***x***_*i*,*ℓ*_, …, ***x***_*i*,*ℓ*+*L*_|***r***, θ*)/(*L* + 1).

For scanning promoter sequences, we also provide an option for penalizing predictions of the reverse complementary strand, relative to the orientation of the downstream gene. Specifically, a small constant *c* is subtracted from all prediction scores ***s***′ on the reverse complementary strand. Throughout this paper, we use *c* = 0.01.

The scanning process explicitly accounts for aberrant repeats, which may loop out of the repeat array [6]. To this end, we search for putative target boxes with all repeats present in the repeat array, but also all combinations of aberrant repeats removed from the RVD sequence. Due to the normalization of scores by the number of repeats, predictions based on these modified RVD sequences can still be ranked in a common list.

In addition, we provide a box-specific p-value as a statistical measure for the significance of target box predictions. Those p-values may either be computed from a dedicated background set of sequences or from a random sub-sample of the scanned input sequences. In either case, scores are computed for the sub-sequences given the current RVD sequences, then a Gaussian distribution is fitted to those score values, and the p-value for a given score is determined from that Gaussian distribution. While the Gaussian distribution does not perfectly fit the true distribution of score values, it allows for computing p-values with high resolution (as opposed to just using percentages of the scores themselves) and even for score values larger than any of the scores in the random sample. Using this procedure, the mapping from scores to p-values is monotonic, i.e., a larger prediction score results in a smaller p-value. Scanning promoters of a large number of genes for putative target boxes results in a multiple testing problem, and users may choose to apply a correction method of their choice controlling for family-wise error rate or false discovery rate. As a rough guideline under the assumption that promoters of tens of thousands genes are scanned for target boxes, p-values below 10^−6^ may be promising candidates for further inspection.

### Genome-wide predictions and filtering

We use PrediTALE for genome-wide prediction in the genome of *Oryza sativa* Nipponbare (MSU7, http://rice.plantbiology.msu.edu/pub/data/Eukaryotic_Projects/o_sativa/annotation_dbs/pseudomolecules/version_7.0/all.dir/all.chrs.con). We make predictions for each TALE of 3 *Xoo* strains and 10 *Xoc* strains. In order to confirm that the predicted target boxes might indeed be bound by the respective TALE, we use the above-mentioned RNA-seq data to determine if there are differentially transcribed regions around a putative target box. For each of the top 100 predictions, we search ± 3000 bp around the predicted site for regions of at least 400 bp that are differentially expressed. Specifically, we count the number of mapped reads for each 400 bp window in replicates of treatment and control. Counts are then normalized relative to the total number of reads within each library, and replicates are averaged separately for treatment and control. Here, we consider a region as differentially expressed if the mean normalized number of reads after infection (treatment) is at least 2-fold larger than the mean normalized number of reads in the control experiment. If several, adjacent 400 bp regions meet this criterion, those are joined to a common, longer region.

This procedure is implemented in a tool called DerTALE. As input, DerTALE expects genomic positions, i.e., the position of predicted target boxes, and BAM files of mapped reads for replicates of treatment and control. Region width, thresholds and averaging methods may be adjusted by user parameters.

For each predicted target box, a profile output is generated if there is at least one differential expressed region with a minimum length of 400 bp that does not overlap the target box, or if it overlaps, the differential region starts or ends at most 50 bp upstream or downstream of the target box.

The obtained profiles may be visualized using an auxiliary R script. In addition to the profile data, this R script requires annotations data of already known transcripts in gff3 format. By this means, users may then investigate whether the predicted binding site may activate the transcription of a gene that has not been annotated yet. Here, we use the MSU7 annotation (http://rice.plantbiology.msu.edu/pub/data/Eukaryotic_Projects/o_sativa/annotation_dbs/pseudomolecules/version_7.0/all.dir/all.gff3).

For differentially expressed regions without annotated MSU7 transcript, we searched for similar sequences using blastx of NCBI BLAST+ version 2.7.1 ftp://ftp.ncbi.nlm.nih.gov/blast/executables/blast+/LATEST/ and choose the non-redundant protein sequence (nr) database. In cases, where we did not receive a convincing hit, we additionally compared sequences with blastn against the reference RNA sequences (refseq_rna) database.

### Implementation and scanning speed-up

For scanning large input sequences, e.g., complete genomes of host plant species, an acceptible runtime is essential. Since the parameters at each position of the proposed model depend on the RVD sequence of the TALE of interest but do not include dependencies between different nucleotides of a putative target box, we may convert the model given a fixed TALE RVD sequence into an position weight matrix (PWM) [32, 33]. This allows for a quick computation of prediction scores that may be formulated as the position-wise sum of values stored in the TALE-specific PWM model. We further speed-up the scanning process by pre-computing indexes of overlapping *k*-mers in the same manner as proposed for the TALENoffer application earlier [34].

### Evaluation of prediction results

We compare the performance of the approach presented in this paper to those of established tools for predicting TALE target sites, namely Target Finder [14], Talvez [16], and TALgetter [18], based on RNA-seq data after inoculation with different *Xoo* and *Xoc* strains described above.

To this end, we collect the promoter sequences of all transcripts based on the MSU7 assembly and gene models [35] available from http://rice.plantbiology.msu.edu/pub/data/Eukaryotic_Projects/o_sativa/annotation_dbs/pseudomolecules/version_7.0/all.dir/. We consider as promoter the sequence spanning from 300 bp upstream of the transcription start site to 200 bp downstream of the transcription start site or the start codon, whichever comes first, as proposed before [18]. We then run each of the tools using default parameters on the extracted promoter sequence providing the RVD sequences of the TALEs present in the respective *Xanthomonas* strain (cf. S1 Data). Predictions in promoters of different transcripts belonging to the same gene are merged by considering only the prediction yielding the best prediction score.

Assessment of prediction performance based on *in-planta* inoculation experiments with *Xanthomonas* strains harboring multiple TALEs has the inherent complications that i) putative target genes cannot be attributed to one specific TALE based on the RNA-seq data alone and ii) genes showing increased expression after inoculation may either be regulated directly by a TALE binding to their promoter or indirectly via other, regulatory target genes. Hence, we define *true positives* as those genes that have a predicted target box in their promoter and are also up-regulated after inoculation with the respective *Xanthomonas* strain relative to control as derived from RNA-seq data. By contrast, we cannot clearly define *false negatives*, since genes that are up-regulated after inoculation but do not contain a predicted target box in their promoter could be indirect target genes. *False positives*, in turn, would be genes with a predicted target box in their promoter that are *not* up-regulated after *Xanthomonas* inoculation.

A further issue hampering performance assessment by standard methods like receiver operating characteristic (ROC) [36] or precision-recall (PR) curves [37, 38] is that for two of the tools considered (Target Finder and Talvez), none of the reported prediction scores is comparable between different TALEs, especially TALEs of different lengths. Hence, we decide to use varying cutoffs on the number of predicted target genes *per TALE* to establish a common ground for comparing all four approaches.

Following these considerations, we collect for each of the four approaches the number of true positive predictions (TPs) for cutoffs on the number of predictions per TALE from 1 (i.e., the top prediction) to 50. We then plot for each approach the number of true positives against this cutoff to obtain a continuous picture of its prediction performance. In addition, we collect for the same cutoffs the number of TALEs with at least one predicted target gene among the true positives.

The area under these curves may serve as a further measure of general prediction performance in analogy to, for instance, the area under the ROC curve.

Finally, we compare the TPs at distinct cutoffs (1, 10, 20, 50) between the four tools. For a specific cutoff, we collect the TPs (or, in analogy, number of TALEs with at least one predicted target) for each of the four tools. Statistical significance of the differences in observed TPs is then assessed by a Quade test [39] using the quade.test function in R [40] and pairwise comparisons are performed by the post-hoc test implemented in function quadeAllPairsTest of the PMCMRplus R-package [41].

In addition, we obtain promoter sequences of five plant species to test PrediTALE for pathosystems beyond *Xanthomonas oryzae*—rice. To this end, we download genome sequences and gene annotations from phytozome (https://phytozome.jgi.doe.gov) for cassava (*Manihot esculenta*, v7.0, [42]), sweet orange (*Citrus sinensis*, v1.1, [43]), cotton (*Gossypium raimondii*, v2.1, [44]), and from solgenomics (https://solgenomics.net) for tomato (*Solanum lycopersicum*, ITAG3.20, [45]) and pepper (*Capsicum annuum* CM334, v1.55, [46]). For these plant species, we consider as promoter the sequence from 300 bp upstream of the annotated transcription start site to the start codon to be less dependent on the exact annotation of transcription start sites.

### Availability

PrediTALE is available as a web-application based on Galaxy at http://galaxy.informatik.uni-halle.de. Both PrediTALE and DerTALE are available as command line application from http://jstacs.de/index.php/PrediTALE and have also been integrated in AnnoTALE 1.4. Source code is available from https://github.com/Jstacs/Jstacs in packages projects.tals.linear, projects.tals.prediction, projects.tals.training, and projects.tals.rnaseq, where also provide an XML representation of the trained model at projects.tals.prediction.preditale_quantitative_PBM.xml. The parameters of the PrediTALE model will be adapted as additional training data become available in the future, while we will preserve a history of PrediTALE models to assure reproducibility. PrediTALE and DerTALE will also be maintained as part of the AnnoTALE suite.

## Results/Discussion

### Benchmarking PrediTALE against previous approaches

In this section, we benchmark the predictions of PrediTALE against those made by one of the previous approaches, namely Target Finder [14], Talvez [16], and TALgetter [18].

To this end, we consider different *Xanthomonas oryzae* pv. *oryzae* (*Xoo*) and *Xanthomonas oryzae* pv. *oryzicola* (*Xoc*) strains for which we have an experimental support of up-regulated genes in *Oryza sativa* after infection based on RNA-seq data. Specifically, we consider the *Xoo* strains ICMP 3125^T^, PXO142 and PXO83 with in-house RNA-seq data available, and the *Xoc* strains B8-12, BLS256, BLS279, BXOR1, CFBP2286, CFBP7331, CFBP7341, CFBP7342, L8 and RS105 based on public RNA-seq data [9]. For the TALEs from the repertoires of these three *Xoo* and ten *Xoc* strains, we determine target gene predictions for each of the previous approaches and for PrediTALE. Predicted target genes are ranked by the corresponding prediction scores of the different approaches per TALE.

**Fig 1:**
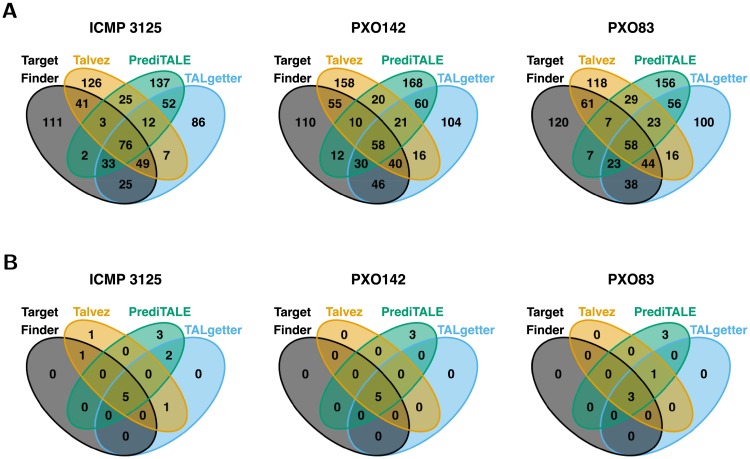
Venn diagrams of predictions of the four approaches considered. (A) For each *Xoo* strain and each approach, we consider the set of target genes obtained as the union of the top 20 predictions per TALE. For *Xoo* ICMP 3125^T^ harboring 17 TALEs, this results in a total number of 340 raw predictions per approach, where the actual number in the diagram may be slightly lower if two TALEs are predicted to target the same gene. For *Xoo* PXO142 (19 TALEs), we obtain 380 raw predictions and for *Xoo* PXO83 (18 TALEs), we obtain 360 raw predictions per approach. (B) Venn diagrams of the subsets of genes from sub-figure A that are also up-regulated according to RNA-seq data.

First, we study the overlaps between the sets of predicted target genes per approach to investigate how strongly predictions are affected by conceptual differences of these approaches. In Fig 1A, we show Venn diagrams of predicted target genes for the three *Xoo* strains based on the top 20 predictions per TALE, while the corresponding diagrams for the ten *Xoc* strains are available as S1 Fig. In general, we observe a substantial number of unique predictions for each of the four approaches, but especially for Talvez and PrediTALE. By contrast, the overlapping predictions between all four approaches amount to less than a quarter of the total predictions per approach. This demonstrates that prediction results strongly depend on the employed approach. However, prediction accuracy cannot be assessed without an experimental knowledge about genes that are up-regulated *in planta* upon *Xanthomonas* infection.

RNA-seq data for the three *Xoo* strains including previously unpublished data for PXO83, have been collected 24 hours after infection. Collection at this early time point has the advantage that the number of secondary targets, i.e., genes that are up-regulated as a secondary effect of direct TALE targets with regulatory function, should still be low. However, as the infection might not be fully established, yet, the variation between replicates and, hence, the number of significantly differentially expressed genes based on standard FDR-based criteria is rather low (cf. Table A in S2 Text). As we aim at sensitivity for the benchmark study, i.e., we want to avoid predictions to be erroneously counted as false positives, we consider genes as differentially up-regulated if they obtain an uncorrected p-value below 0.05 and are at least 2-fold up-regulated in this case, which results in 43 (PXO142) to 107 (ICMP 3125^T^) differentially up-regulated genes.

In case of the ten *Xoc* strains, RNA-seq data have been recorded 48 hours after infection. Here, infection should be fully established, but we expect a substantial number of secondary targets to be up-regulated already. Hence, we resort to rather standard thresholds with a FDR-corrected *q* − *value* < 0.01 and log fold change greater than 2 in this case. Notably, this still results in a larger number of differentially up-regulated genes (cf. Table B in S2 Text) than for the *Xoo* strains with numbers between 202 (CFBP2286) and 672 (L8).

Given these up-regulated genes as a *ground truth*, we may now count predictions of TALE target boxes in promoters of up-regulated genes as *true positives*, and predictions without observed up-regulation as *false positives*. In Fig 1B, we plot Venn diagrams of the true positives among the top 20 predictions of all four approaches. Notably, we find that the intersection of the predictions of all four approaches constitutes (one of) the largest set(s) in each of the three Venn diagrams. Among the predictions that are unique to one of the four approaches, we consistently find the largest number of true positive predictions for PrediTALE, which indicates the utility of our novel approach. Turning to the ten *Xoc* strains (S2 Fig), we again find the same tendency with regard to the predictions overlapping among all four approaches. However, the number of true positives among the unique predictions shows a less clear picture with a slight advantage towards Talvez, while predictions of PrediTALE often overlap with TALgetter and/or Target Finder. Together, the Venn diagrams for the *Xoo* and *Xoc* strains also illustrate why it is generally beneficial to complement *in silico* TALE target predictions with experimental data about gene regulation.

The results presented so far strongly depend on the thresholds of the ranks of the target predictions but also on the thresholds applied to the RNA-seq data. To address the former problem, we aim at an assessment of target predictions over all rank thresholds, while we will handle the latter by separate evaluations applying different criteria to the RNA-seq data.

As detailed in section “Evaluation of prediction results”, standard performance measures like the area under the ROC curve [36] or the area under the precision-recall curve [37, 38] are inappropriate under this setting. Briefly, we cannot attribute an up-regulated gene to a specific TALE from the TALE repertoire of the strain under study. In addition, genes that are up-regulated in the RNA-seq experiment might also be due to secondary effects of TALE targets, due to general plant response to the bacteria, or due to other classes of effector proteins. Thus, we may not consider up-regulated genes *without* a matching prediction of a TALE target box in their promoter as *false negatives*. Hence, we decide to compare the performance of different approaches by means of the number of *true positive* predictions at different rank cutoffs, i.e., considering the top *N* predicted target genes of each approach.

**Fig 2:**
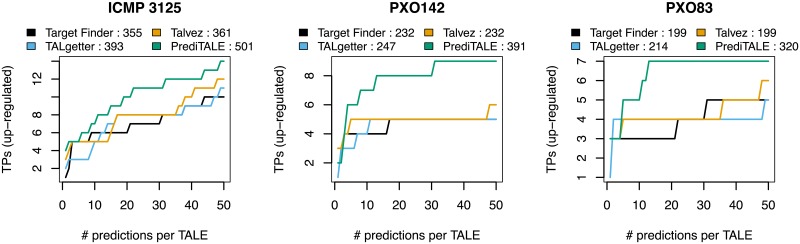
Performance evaluation on the level of target genes for three *Xoo* strains. For each approach, we plot the number of predicted target genes that are also up-regulated in the infection (true positives, TPs) against the number of predicted target sites per TALE. In the legends, we further report the areas under the curves after the name of the individual approaches.

In Fig 2, we plot the number of true positives for the three *Xoo* strains and each of the four approaches against the total number of predictions per TALE, considering only the highest-ranking prediction up to 50 target predictions per TALE, which we consider a reasonable cutoff under the scenario of manual inspection. In addition, we compute the area under this curve as an overall performance statistic across all rank cutoffs. For all three *Xoo* strains, we find that PrediTALE dominates the other three tools for rank cutoffs of 5 and above. For lower rank cutoffs, the ranking of tools is less clear, but PrediTALE still yields—for instance—the largest number of true positive predictions on rank 1 for two of the three strains. In the ranking with regard to the area under the curve (AUC), we find that PrediTALE again yields the best overall performance among all four approaches.

**Fig 3:**
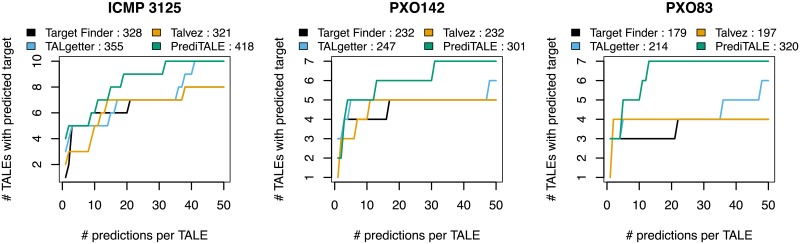
Performance evaluation on the level of TALEs for three *Xoo* strains. For each approach, we plot the number of TALEs with at least one predicted target gene that is also up-regulated in the infection against the number of predicted target sites per TALE.

We take a different perspective on prediction results by assessing prediction performance on the level of TALEs. Specifically, we count the number of TALEs with at least one true positive target prediction for the same rank cutoffs as before. Again, PrediTALE identifies targets for a larger number of TALEs than the other approaches for the majority of rank cutoffs (Fig 3). However, we see notable differences between the different *Xoo* strains, where PrediTALE is able to identify putative targets for 10 of the 17 TALEs of ICMP 3125^T^, but only for 7 out of 19 TALEs for PXO142 and for 7 out of 18 TALEs for PXO83. As ICMP 3125^T^ has also been the strain with the largest number of differentially up-regulated genes (cf. Table A in S2 Text), the lower number of TALEs in PXO142 and PXO83 with a predicted target might be due to a different progression of the *Xanthomonas* infection.

**Fig 4:**
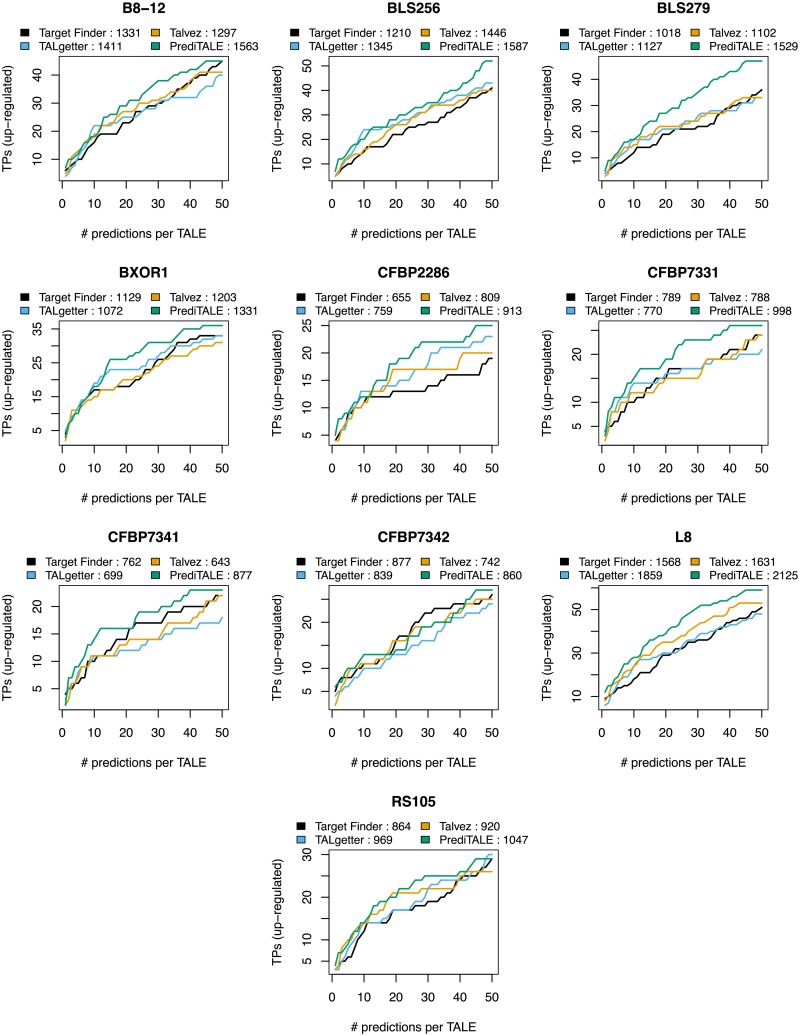
Performance evaluation on the level of target genes for 10 *Xoc* strains. For each approach, we plot the number of predicted target genes that are also up-regulated in the infection (true positives, TPs) against the number of predicted target sites per TALE.

We further summarize the data behind Figs 4 and 3 in Tables C and D in S2 Text, where we also report the average ranks of the four approaches across all three *Xoo* strains.

For sake of completeness, we also evaluate the four approaches for differentially up-regulated genes after *Xoo* infection based on the same FDR-based thresholds as for the *Xoc* experiments (S3 and S4 Figs).

Although it has been shown that TALEs may activate transcription in both strand orientations relative to the transcription start site (TSS) of target genes [12, 13], a preference for the forward orientation has been postulated [13]. This is reflected by the strand penalty of PrediTALE, but no similar parameter exists for the previous approaches. Hence, above comparison might be perceived as partially unfair in favor of PrediTALE. For this reason, we repeat the benchmarking after restricting the predictions of all four approaches to a forward orientation relative to the TSS (S5 and S6 Figs). While the restriction to the forward strand has an effect on the number of target genes and TALEs with at least one true positive target, PrediTALE still yields an improved performance compared with the previous approaches over a wide range of rank cutoffs and, hence, achieves the largest AUC value of the four approaches in all cases.

For the ten *Xoc* strains, we find an improved prediction performance for PrediTALE as well. On the level of true positive target genes (Fig 4), PrediTALE yields the largest number of true positives for a rank cutoff of 1 for seven of the ten *Xoc* strains (cf. Table I in S2 Text). We also find an improved performance for the majority of the remaining rank cutoffs and *Xoc* strains. This improvement is especially pronounced for strains *Xoc* BLS279, CFBP7331, CFBP7341, and L8, whereas PrediTALE performs similar to or slightly worse than at least one of the previous approaches for *Xoc* CFBP7342 and RS105. For the remaining strains (B8-12, BLS256, BXOR1, CFBP2286), the improvement by PrediTALE is either rather small or mostly restricted to rank cutoffs of 20 or larger. This is also reflected by the areas under the curves, where PrediTALE yields the largest areas for B8-12, BLS256, BLS279, BXOR1, CFBP2286, CFBP7331, CFBP7341, L8, and also RS105, but nor for CFBP7342. Results are largely similar on the level of TALEs with at least one true positive predicted target (S7 Fig), where PrediTALE yields the largest area under the curve for the same strains.

To obtain a more condensed overview on the results for the *Xoc* strains, we finally compute the average performance ranks across all ten *Xoc* strains for each of the four approaches and fixed rank cutoffs of 1, 10, 20, and 50, and for the area under the curve both on the level of target genes and on the level of TALEs (Table 1 and Table I and J in S2 Text). For all rank cutoffs and the area under the curve, we observe that PrediTALE yields the best average rank with values betwen 1.1 and 1.5. We further assess the statistical significance of differences between the different tools by a Quade test, and the pairwise differences between tools by the associated post-hoc test (see Methods). This assessment is partly limited by the fact that pairs of *Xoc* strains may have identical TALEs in their TALEomes, which also means that the performance values of those strains are not truly independent. However, we did not find a clear relationship between the similarity of performance values obtained for the different strains and the similarity of the corresponding TALEomes. For this reason, we consider this dependency rather mild and favor this limited statistical assessment over the complete lack of it.

**Table 1:** Testing the significance of differences in prediction performance.

measure	TF	Tg	Tv	PT	Quade	Tg/TF	Tv/TF	Tv/Tg	PT/TF	PT/Tg	PT/Tv
Genes R1	1.8	3.1	2.4	1.5	**	—	-			+++	++
Genes R10	3.6	2.5	1.6	1.5	***	+	+++	+	+++	+	
Genes R20	3.3	2.1	2.9	1.3	***	+++	+		+++	++	+++
Genes R50	2.3	3	3	1.1	**				+++	+++	+++
Genes AUC	3.1	2.8	3	1.1	***				+++	+++	+++
TALEs R1	1.8	3.1	2.4	1.5	**	—	-			+++	++
TALEs R10	3.5	2.1	1.6	1.5	***	+	+++	++	+++	+	
TALEs R20	3.5	1.8	2.6	1.2	***	+++	+	-	+++		+++
TALEs R50	2.6	2.6	2.8	1.4	**				++	++	+++
TALEs AUC	3.4	2.6	2.8	1.1	***	++	+		+++	+++	+++

TF: Target Finder; Tg: TALgetter; Tv: Talvez; PT: PrediTALE. For each tool and each measure (TALEs/Genes; rank cutoff), we report the average performance rank per tool, the significance of the Quade test (*:< 0.05; **:< 0.01; ***:< 0.001), and the significance of the pairwise comparison in a post-hoc test. Here, ‘+’ and ‘-’ indicate that the first tool has gained a significantly better or worse performance than the second one, respectively. The number of symbols encodes the significance level in analogy to the Quade test.

Consistent with the previous observations, we find that PrediTALE never performs significantly worse then any of the three previous approaches, whereas in many cases it performs significantly better, often with p-values below 0.001 in the post-hoc test. Notable exceptions are a rank cutoff of 1, where PrediTALE does not perform significantly different from Target Finder, a rank cutoff of 10, where PrediTALE does not perform significantly different from Talvez, and on the level of TALEs, a rank cutoff of 20, where PrediTALE does not perform significantly different from TALgetter.

Repeating the same analysis for varied q-value threshold (S8 and S9 Figs, Table K, L, and M in S2 Text), for varied log fold change threshold (S10 and S11 Figs, Table N, O, and P in S2 Text), and for predictions restricted to the forward strand relative to the TSS (S12 and S13 Figs, Table Q, R, and S in S2 Text), benchmarking results are essentially similar to our previous findings. One notable exception is the Quade test for rank 1 predictions restricted to the forward strand (Table S in S2 Text), which is no longer significant. This means that none of the approaches studied yields significantly better rank 1 predictions than any other under this scenario.

Although the focus of this manuscript is on target predictions for TALEs from *X. oryzae* strains, PrediTALE may as well be applied to TALEs from other *Xanthomonas* species. To illustrate this, we perform promoterome-wide scans for putative target boxes of TALEs from five additional *Xanthomonas* species and corresponding host plants for which virulence targets have been published previously. We find the known targets of these five TALEs on rank 1 or 2 of the corresponding PrediTALE predictions (Table 2 and S6 Table). Interestingly, the top prediction of PrediTALE for AvrBs3 in pepper is a different target (transcription factor bHLH137, CA06g21040) than the well described target (transcription factor UPA20, CA03g22700) [47].

**Table 2:** Known virulence targets of five strains from different *Xanthomonas* species and the ranks among the PrediTALE predictions in the promoteromes of their host plant species for the corresponding TALEs.

Species/strain	Host plant	TALE	Target gene (ID)	Rank
*X. axonopodis* pv. *manihotis* Xam668	Cassava	TAL20_*Xam*668_	MeSWEET10a [48] (Manes.06G123400.1)	2
*X. citri* subsp. *malvacearum* XcmH1005	Cotton	Avrb6	GhSWEET10 [49] (Gorai.008G209000)	2
*X. gardneri*	Tomato	AvrHah1	bHLH3 [50] (Solyc03g097820)	1
*X. citri* subsp. *citri* Xcc306	Sweet orange	pthA4	CsLOB1 [51] (orange1.1g026556m)	1
*X. euvesicatoria*	Pepper	AvrBs3	UPA20 (bHLH TF) [47] (CA03g22700)	2

Summarizing the benchmark studies, we find i) that PrediTALE produces several unique predictions that might not have been considered based on previous approaches, ii) although low in absolute terms, the number of true positives among these predictions is often larger than for the previous aproaches, and iii) an assessment of the performance of PrediTALE across a wide range of rank cutoffs demonstrates that in most of the cases the application of PrediTALE yields a larger number of true positive target predictions than any of the three previous approaches. However, we also observe true positive predictions of one of the previous approaches that would be missed by PrediTALE. A general recommendation would be to use the union of the predictions of all four tools when aiming for sensitivity, i.e., to recognize as many true positives as possible. Aiming at precision instead, i.e., maximizing the fraction of true positives in the predictions considered, our results indicate that using either only PrediTALE predictions or predictions in the intersection of all four approaches would be recommended.

### Evaluating different aspects of the PrediTALE model

**Fig 5:**
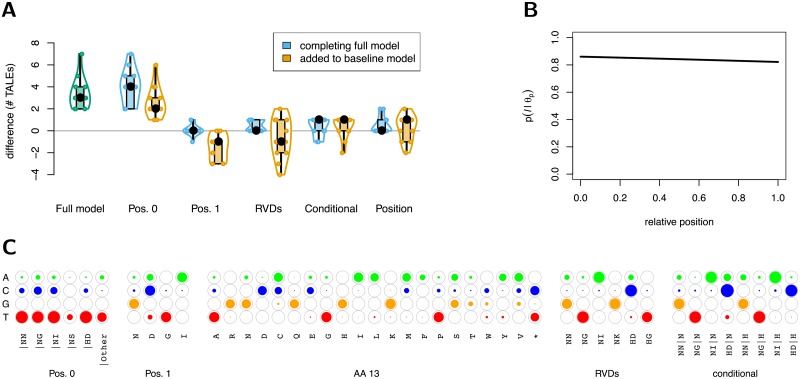
Assessment of different aspects of the PrediTALE model. (A) Comparing the full model to the baseline model using only specificities based on AA 13 of an RVD and independent parameters for position 0. For each subset of features, we additionally compare the case where i) features are completing the full model and ii) features are added to the baseline model. We show violin plots of the number of TALE with at least one true positive target using at most 20 predictions per TALE (cf. Fig 3) including individual points for all *Xoo* and *Xoc* data sets. (B) Position distribution of the full model, which is basically a straight line decreasing only marginally to the end of a sequence. (C) Parameters of the full PrediTALE model represented by circles filled to a degree proportional to specificity parameters.

Having established that PrediTALE often yields an improved performance compared with previous approaches, we investigate in the following, which aspects of the PrediTALE model contribute to which extent to the performance of the full PrediTALE model. To this end, we first consider a baseline model for which we define the sets $R_{0}$, $R_{1}$, $R_{2}$ and $R_{3}$ as empty sets, and set the position-dependent term *p*(*ℓ*|***θ***_*p*_) to a uniform distribution. Starting from this baseline model, we then individually restore each individual set and the position-dependent term to its original value, and record the difference in the observed performance. Reciprocally, we consider the full model and determine the difference of its performance to a model where only one of the individual sets is defined as empty or the position-dependent term is set to uniform. In Fig 5, we present the results of this analysis, again considering the number of TALEs with at least one true positive prediction based on the top 20 predictions per TALE, while the respective results with regard to the total number of true positive target genes are shown in S14 Fig. As a reference, we also include the difference in performance of the full model compared with the baseline model.

We find that the results of the two perspectives (adding a feature to the baseline model vs. completing the full models) are contradictory. While some of the features even reduce performance when added to the baseline model (separate specificities for position 1, $R_{2}$; specificities for individual RVDs, $R_{1}$), all features increase performance either on the level of TALEs or target genes when completing the full model. The specificities at position 0 dependending on the first RVD ($R_{0}$) are a notable exception. Here we observe an improvement of performance in either case, which is also substantially greater than for any of the other features.

However, this effect may not only be attributed to the specificities at position 0 being modeled depending on the first RVD. Inspecting the specificity parameters of the full model (Fig 5C) and comparing these to those of the baseline model, the baseline model with $R_{0}$ restored, and the full model with $R_{0}$ set to the empty set (S15 Fig), we find complex interactions among the specificity parameters. As this study has been conducted with a large number of independent restarts of the procedure optimizing model parameters, this is unlikely an effect of the optimization getting stuck in local optima. Rather the objective function (difference between observed quantities and prediction scores) appears to skew some of the remaining model parameters to achieve its optimum if the model is lacking the conditional specificities at position 0. Nonetheless, these results indicate that the inclusion of specificities at position 0 depending on the first RVD is an essential ingredient of the PrediTALE model. Currently, this aspect is limited by the corresponding training data from [23] and, hence, it might be a worthwhile perspective to quantitatively investigate this dependency for further RVDs in the future.

In addition, the specificity parameters of the PrediTALE model may also contain interesting patterns *per se*. For instance, we find that base preference at position 0 given RVD “NS” at position 1 is less clear than for other RVDs, where the known target box of TalC (TalBS1) harboring “NS” at position 1 is preceeded by a ‘C’ in the promoter of Os11N3 [52]. The UPA target box of AvrBs3 in pepper has an ‘A’ at position 1 [4], although the first RVD of AvrBs3 is “HD”, which complies with the specificity of ‘D’ at position 1 being shifted towards base ‘A’ relative to the general preference of RVDs with AA 13 equal to ‘D’ being ‘C’.

Finally, we consider the position-dependent term of the full model (Fig 5B), and find that it is much simpler than allowed by the mixture of two logistic functions, corresponding to a straight, slightly decreasing line. In contrast to the specificity parameters, the position-dependent term seems to be largely independent of the specificity features (cf. S15 Fig).

As all features contribute, at least slightly, to the performance of the full PrediTALE model, we consider this model in the remainder of this manuscript.

### PrediTALE predicts novel putative target genes

As we have seen from Fig 1B, putative target genes with up-regulation after *Xoo* infection are often found in the intersection of the predictions of all four approaches. In addition, PrediTALE predicts several putative target genes of TALEs from the three *Xoo* strains that might have been neglected using one of the previous tools. In the following, we scrutinize the predictions for the *Xoo* strains with a focus on novel predictions, while we give a complete list of top 20 predictions of all four approaches including the ten *Xoc* strains in S3 Table.

In Table 3, we collect further information about those target genes including the corresponding log fold change and prediction ranks for all four approaches.

**Table 3:** Putative TALE target genes that are among the top 20 predictions per TALE for any of the four approaches.

Gene	lfc	Target Finder	Talvez	Talgetter	PrediTALE	annotation
**ICMP 3125^T^**						
Os04g43730	5.762	TalES1 (9)	TalAR13 (19); TalES1 (10)	TalAR13 (564); TalES1 (67)	TalAR13 (472); TalES1 (108)	OsWAK51
Os02g06670	3.815	TalBA8 (1)	TalBA8 (2)	TalBA8 (1)	TalBA8 (1)	retrotransposon protein
Os09g29820	2.819	TalAR13 (2)	TalAR13 (1)	TalAR13 (3)	TalAR13 (2)	bZIP transcription factor
Os03g51760	2.734	TalAD22 (21)	TalAB16 (407); TalAD22 (209)	TalAD22 (17)	TalAD22 (9)	OsFBX109—F-box protein
Os04g05050	2.221	TalAB16 (490)	NA	TalAB16 (63)	TalAB16 (11); TalAH11 (824)	pectate lyase
Os01g40290	1.894	TalAA15 (3)	TalAA15 (12)	TalAA15 (1)	TalAA15 (1)	expressed protein
Os05g45070	1.704	NA	NA	TalAO15 (214)	TalAF17 (559); TalAO15 (15)	harpin-induced protein 1
Os11g26790	1.695	TalAH11 (3)	TalAH11 (1)	TalAH11 (1); TalAQ14 (559)	TalAH11 (1)	dehydrin
Os06g03710	1.591	TalES1 (44)	TalES1 (81)	TalES1 (41)	TalES1 (19)	DELLA protein SLR1
Os03g03034	1.295	TalAO15 (404); TalAQ14 (125)	TalAO15 (396); TalAQ14 (9)	TalAB16 (600); TalAO15 (566); TalAQ14 (15)	TalAB16 (220); TalAO15 (556); TalAQ14 (32)	flavonol synthase
Os01g73890	1.079	TalBM2 (3)	TalBM2 (14)	TalBM2 (2)	TalBM2 (1); TalET1 (477)	transcription initiation factor IIA gamma
Os10g28240	0.918	TalAR13 (71)	TalAR13 (47)	TalAR13 (16)	TalAR13 (6)	calcium-transporting ATPase
Os09g07460	0.746	TalBA8 (88)	TalBA8 (17)	TalBA8 (48)	TalBA8 (22)	kelch repeat protein
**PXO142**						
Os02g49350	5.163	TalBH2 (1)	TalBH2 (2)	TalBH2 (5)	TalBH2 (8)	plastocyanin-like
Os03g09150	2.530	NA	NA	TalBK2 (805)	TalBH2 (4); TalBK2 (239)	pumilio-family RNA binding
Os11g31190	2.514	TalAN15 (681)	TalAE16 (530); TalBH2 (848)	TalAQ15 (660); TalBH2 (144)	TalBH2 (3)	SWEET14 (nodulin MtN3)
Os09g29820	2.272	TalAR14 (1)	TalAR14 (2)	TalAR14 (1)	TalAR14 (3)	bZIP transcription factor
Os03g51760	1.368	TalAD23 (77)	TalAD23 (288)	TalAD23 (48)	TalAD23 (13); TalAS12 (421)	OsFBX109—F-box protein
Os01g40290	0.887	TalAA16 (3)	TalAA16 (7)	TalAA16 (1)	TalAA16 (1)	expressed protein
Os06g29790	0.833	TalAO16 (17)	TalAO16 (11)	TalAO16 (3)	TalAO16 (4); TalAP15 (799)	phosphate transporter 1
Os07g06970	0.824	TalAP15 (1); TalAQ15 (521)	TalAP15 (1); TalAQ15 (319)	TalAP15 (1); TalAR14 (563)	TalAI17 (889); TalAP15 (1)	HEN1
**PXO83**						
Os09g29820	2.82	TalAR3 (1)	TalAR3 (2)	TalAR3 (1)	TalAR3 (5)	bZIP transcription factor
Os02g06670	2.74	TalBA2 (1)	TalBA2 (2)	TalAR3 (996); TalBA2 (1)	TalAR3 (83); TalBA2 (1)	retrotransposon protein
Os03g51760	1.91	TalAD5 (77)	TalAB5 (407); TalAD5 (288)	TalAD5 (48)	TalAD5 (13)	OsFBX109—F-box protein
Os04g19960	1.70	NA	TalAN3 (668); TalAP3 (365)	TalAP3 (588)	TalAC5 (1); TalAN3 (846)	retrotransposon protein
Os04g05050	1.62	TalAB5 (490)	TalAP3 (931)	TalAB5 (63)	TalAB5 (11)	pectate lyase
Os07g06970	1.40	TalAP3 (1)	TalAP3 (1); TalAQ3 (512)	TalAP3 (1); TalAR3 (988)	TalAP3 (1)	HEN1
Os03g03034	1.18	TalAO3 (404); TalAQ3 (70)	TalAO3 (396); TalAQ3 (2)	TalAB5 (600); TalAO3 (566); TalAQ3 (5)	TalAB5 (220); TalAO3 (556); TalAQ3 (5)	flavonol synthase

For each *Xoo* strain, we list the gene ID (MSU7) and the log fold change (lfc) in the corresponding RNA-seq experiment. For each of the four approaches, we further list the TALE(s), for which a gene has been predicted as a target and in parentheses the corresponding prediction rank. An “NA” entry for a combination of gene and prediction approach indicates that this gene has not been among the top 1000 predictions for any TALE.

The target genes in the intersections of the predictions of all four approaches comprise several well known targets. For instance, Os09g29820 (OsTFX1), a bZIP transcription factor, is targeted by TALEs from class TalAR with members in all three *Xoo* strains (S16 Fig) and has been proposed as a TALE target early [5, 53].

Os01g73890 (TFIIA*γ*) [5], that has been shown to promote TALE function [54], is targeted by TalBM2 in ICMP 3125^T^. In concordance to TalBM class members missing in PXO142 and PXO83, Os01g73890 shows no up-regulation in these two strains. Os07g06970 (HEN1) has also been among the first TALE target genes proposed [5] and is targeted by TalAP members present in all three *Xoo* strains, but falls below the threshold on the log fold change by a small margin in ICMP 3125^T^ (S17 Fig). Os01g40290 [5], an expressed protein without annotated function, Os06g29790 [18], a phosphate transporter, and Os11g26790 [16] (RAB21), a dehydrin that has been shown to play a role in drought tolerance related to pathogen infection [55], have also been predicted in previous studies.

In addition, we find putative target genes in the intersection that have not been reported before: Os02g06670, a retrotransposon protein, is predicted as a target of TalBA8 and TalBA2 in ICMP 3125^T^ and PXO83, respectively, whereas PXO142 lacks a TalBA member. Nonetheless, Os02g06670 is up-regulated after PXO142 infection, although to a lesser degree than in the other two strains (cf. S17 Fig). Os02g49350, a plastocyanin-like protein, is strongly up-regulated only in PXO142 and predicted as a target of TalBH2, where class TalBH is exclusive to PXO142 among the strains studied.

Finally, we find several putative target genes that have been predicted only by a subset of approaches: For ICMP 3125^T^, Os04g43730 [56] (OsWAK51) is among the top 20 predictions for TalES1 only for Target Finder and Talvez. In turn, PrediTALE predicts Os06g03710 (DELLA protein SLR1) as a TalES1 target on rank 19, which appears on later ranks for the other approaches. Os04g43730 is induced more strongly than Os06g03710 and exclusively in ICMP 3125^T^, which renders this the more likely target. Os03g51760 [16] (OsFBX109) is among the top 20 predictions for TalAD members only for PrediTALE. Due to variations in their RVD sequence, TALgetter has this in the top 20 predictions only for TalAD22 in ICMP 3125^T^, but not for the other strains. As Os03g51760 is clearly up-regulated after infection with any of the three *Xoo* strains (S17 Fig), this is likely a true TalAD target.

Talvez and TALgetter have Os03g03034, annotated as a flavonol synthase, among their top 20 predictions for TalAQ members in ICMP 3125^T^ and PXO83, while this gene is among the top 20 predictions of PrediTALE only for TalAQ3 in PXO83 due to differences in RVD sequence. In PXO142, TalAQ15 is annotated as a pseudo gene and this pattern is also reflected by the RNA-seq data. Os03g03034 has been proposed to be a TALE target before [5, 56].

Os04g05050 [16, 56], annotated as a pectate lyase, is only among the top 20 predictions of PrediTALE in ICMP 3125^T^ (TalAB16) and PXO83 (TalAB5), whereas this gene is ranked substantially lower (rank 83) for TalAB8 from PXO142 by PrediTALE as well. From the RNA-seq data, we find that Os04g05050 is up-regulated in all three *Xoo* strains, although the level of up-regulation is lower for PXO142 than for the other two strains.

Os05g45070, annotated as hairpin-induced protein 1, is predicted only by PrediTALE as an alternative target of TalAO15 in ICMP 3125^T^ and shows clear up-regulation only after infection with this *Xoo* strain. Os10g28240 [16], a calcium transporting ATPase, is predicted by TALgetter and PrediTALE as target of TalAR13 of ICMP 3125^T^ but, on later ranks, also by the other two approaches, and is up-regulated exclusively after ICMP 3125^T^ infection. Os09g07460 [16], a kelch repeat protein, is only among the top 20 predictions of Talvez for TalBA and on later ranks for the other approaches. This gene is up-regulated only in ICMP 3125^T^, although not strongly.

For PXO142, we find two further putative targets of TalBH2 that are predicted exclusively by PrediTALE: Os03g09150 (pumilio-family RNA binding) is up-regulated in PXO142 but also in PXO83, for which it does not appear among the top 20 predictions of any approach. Os03g09150 has been predicted before as a target of class TalAC [16]. However, PXO142 is lacking members of class TalAC, while Os03g09150 only appears at later ranks for TalAC5 of *Xoo* PXO83. Os11g31190 (Os11N3, OsSWEET14) is a well known target [52, 57], which is predicted here also for TalBH exclusively by PrediTALE due to its ability to adequately handle the aberrant repeat [6] of TalBH2. Os11g31190 is also known to be targeted by TalAC members (previously termed AvrXa7) [53] including TalAC5 in PXO83 and, hence, is strongly up-regulated after PXO83 infection as well. However, in this case all approaches fail to predict this target due to the large number of mis-matches in the target box [6], even accounting for the aberrant repeat in TalAC5.

Instead, another retrotransposon protein (Os04g19960 [58]) is the top prediction of PrediTALE for TalAC5 from PXO83, which is confirmed by RNA-seq data as this gene is strongly up-regulated after PXO83 infection but not after infection with one of the other strains.

In summary, we find several novel putative target genes of which three are highly promising (Os02g49350, Os05g45070, Os03g09150), where two of these (Os05g45070, Os03g09150) are predicted as targets of the respective TALE classes on high ranks exclusively by PrediTALE. Recently, we could experimentally validate the targets Os04g43730 (OsWAK51), Os06g29790 (phosphate transporter), Os03g51760 (OsFBX109), Os03g03034 (flavonol synthase), and Os04g05050 (pectate lyase) by qRT-PCR using a TALE-less strain (Roth X1-8) complemented with individual TALEs [56].

### Orphan TALEs

We also observe from Fig 3 and S7 Fig that for many strains, neither of the approaches considered is able to identify a putative target genes for all TALEs present in their TALEome. We term such TALEs without reasonable target prediction *orphan TALEs*, and we will discuss these in more detail in the following.

More precisely, we call a TALE or a TALE class *orphan* if there is no up-regulated gene among the top 50 predictions of any of the four approaches. Furthermore, we check if this pattern is consistent for the TALEs from a common TALE class across almost all *Xoo* and *Xoc* strains studied.

We find as orphan the TALE classes present in all three *Xoo* strains TalAF, TalAI and TalAN. In addition, TalAG (PXO142, PXO83), TalAL (PXO142), TalAS (PXO142, PXO83), TalBJ (PXO83), TalCA (PXO83), TalET (ICMP 3125^T^), and TalDR (PXO142) are orphan TALE classes in individual *Xoo* strains. The TALEs from class TalAI and TalDR are truncTALEs that are lacking large parts of the C-terminus including the activation domain and, for this reason, do not act as transcriptional activators. TruncTALEs have been found to function as suppressors of resistance mediated by an immune receptor [59].

In the *Xoc* strains, however, TalAF is not orphan as we find putative target genes among the top 50 predictions for the class members present in B8-12 and L8. For TalAZ, we find a target for TalAZ7 from *Xoc* L8, but not for the other 7 *Xoc* strains harboring TalAZ TALEs. In addition, we consider TalCQ1 from BXOR1 and TalCR1 (CFBP7331) and TalCR2 (CFBP7341) as orphan.

Reasons for orphan TALEs could be manifold. First of all, we cannot be sure that these TALEs are indeed expressed by the bacteria and are secreted into the host plant cells. Second, some TALEs might activate target genes slower or to a lesser degree than others and, for this reason, target gene activation might not be detectable, yet, in the RNA-seq experiments, especially at the 24h timepoint chosen for *Xoo*. Third, these TALEs might target specific variants of boxes in promoters of rice lines that are not represented by the *O. sativa* Nipponbare reference genome, or might even target genes in alternative host plants, e.g., grasses in the vicinity of fields where rice is grown. Fourth, these TALEs might target genes that are missing from the current gene annotations of rice. Such targets would be neglected by the current approach to specifically scan promoter sequences of annotated genes for putative TALE boxes. To address the latter issue, we switch to an alternative approach in the following. Here, we perform *genome-wide* scans for putative target boxes instead, and search for differentially expressed regions in the vicinity of putative target boxes predicted anywhere in the reference genome.

### Genome-wide prediction profiles discover potential novel target genes

We perform genome-wide predictions of TALE target boxes in *Oryza sativa* Nipponbare (MSU7) for the 256 *Xoc* TALEs from 10 strains and 54 *Xoo* TALEs from 3 strains and check for differentially expressed regions near the predicted target boxes. Differential expression is based on the mapped RNA-seq data after infection with the respective *Xoo* and *Xoc* strains. Performing genome-wide scans is facilitated by the runtime optimization of the PrediTALE scanning process described in section “Implementation & scanning speed-up”, and we provide a comparison of exemplary running times of genome-wide scans for target boxes of all 28 TALEs of strain *Xoc* BLS256 in Table T in S2 Text.

After infection with *Xoo* strains, 14 TALEs are found to have differentially expressed regions near at least one predicted target box. Table 4 lists the total number of 19 TALE target boxes together with MSU7 gene annotations overlapping the differentially expressed regions. Notably, 15 of these targets have already been reported in subsection “PrediTALE predicts novel putative target genes” when restricting the search to promoter regions of annotated genes. However, for two genes, target boxes from other TALs were predicted in case of genome-wide scan. The expression of the pectate lyase precursor (Os04g05050) was up-regulated by TalAB5 according to promotor prediction, but the genome-wide prediction contains the same gene up-regulated by TalAD22. The same scenario for the phosphate transporter 1 (Os06g29790), which according to promotor predictions is up-regulated by TalAO16 and TalAP15. However, in the genome-wide scans, a target box of TalAH11 was predicted. The genome-wide scan i) does not make use of gene annotations, and ii) could be expected to be more prone to false positive predictions than the restricted search in promoters. Hence, the fact that many predictions re-occur in the genome-wide scan demonstrates the general utility of this approach.

**Table 4:** Genome-wide prediction of *Xoo* TALE targets with PrediTALE.

TALE	Chr	Pos. box	Gene	Annotation	PiP
**ICMP 3125^T^**					
TalAA15	Chr1	22747303	Os01g40290	expressed protein	yes
TalAD22	Chr3	29685233	Os03g51760	OsFBX109—F-box protein	yes
TalAD22	Chr4	2486797	Os04g05050	pectate lyase precursor	TalAB5
TalAH11	Chr6	17129738	Os06g29790	phosphate transporter 1	TalAO16, TalAP15
TalAN14	Chr2	31931460	Os02g52170	expressed protein	no
TalAN14	Chr8	19950534	Os08g32160	oxidoreductase, 2OG-FeII oxygenase	no
TalAR13	Chr10	14685398	Os10g28240	calcium-transporting ATPase	yes
TalAR13	Chr9	18123472	Os09g29820	bZIP transcription factor	yes
TalBA8	Chr2	3353526	Os02g06670	retrotransposon protein	yes
TalBM2	Chr1	42819000	Os01g73890	transcription initiation factor IIA gamma	yes
**PXO142**					
TalAO16	Chr7	22546154	–	–	NA
TalAR14	Chr5	16047774	Os05g27580	wound-induced protein WI12	no
TalAR14	Chr9	18123472	Os09g29820	bZIP transcription factor	yes
TalBH2	Chr11	18174482	Os11g31190	SWEET14 (nodulin MtN3 family)	yes
TalBH2	Chr2	30158664	Os02g49350	plastocyanin-like	yes
**PXO83**					
TalAC5	Chr4	11130506	Os04g19960	retrotransposon protein	yes
TalAP3	Chr7	3434725	Os07g06970	HEN1	yes
TalAQ3	Chr3	1245017	Os03g03034	flavonol synthase/flavanone 3-hydroxylase	yes
TalAR3	Chr9	18123472	Os09g29820	bZIP transcription factor	yes

Genome-wide prediction of *Xoo* TALE targets using PrediTALE filtered for differentially expressed regions within 3000 bp surrounding the target box. For each *Xoo* strain, we list the TALE name, Chromosome number and position of the target box (Pos. box) in *Oryza sativa* Nipponbare genome, and the annotated MSU7 Gene ID and description (if present). In addition, the last column contains the information, whether predictions in promoters (PiP) also report this target.

In addition to those targets reported previously, we find three novel target boxes in the vicinity of differentially expressed regions that overlap annotated genes, including a wound-induced protein and an oxidoreductase. For TalAO16 from PXO142, we find a differentially expressed region next to a predicted target box on chromosome 7 with no annotation in MSU7 (S18 Fig; complete list in S4 Table). For this reason, we extracted the sequence under the differentially expressed region, and first compared it against the NCBI protein database ‘nr’ using blastx but received no matching result. We additionally compared this sequence against the NCBI reference RNA sequences (refseq_rna) using blastn, which resulted in a highly significant hit for XR_001547425.2, a predicted long non-coding RNA.

Upon infection of rice with *Xoc* strains, differentially expressed regions near at least one predicted target box were found for 26 of 28 (B8-12), 28 of 28 (BLS256), 25 of 26 (BLS279), 26 of 27 (BXOR1), 22 of 28 (CFBP2286), 19 of 22 (CFBP7331), 19 of 21 (CFBP7341), 18 of 23 (CFBP7342), 27 of 29 (L8) and 19 of 24 (RS105) TALEs. S5 Table lists all genome-wide predicted targets in the vicinity of differentially expressed regions of these *Xoc* strains.

**Fig 6:**
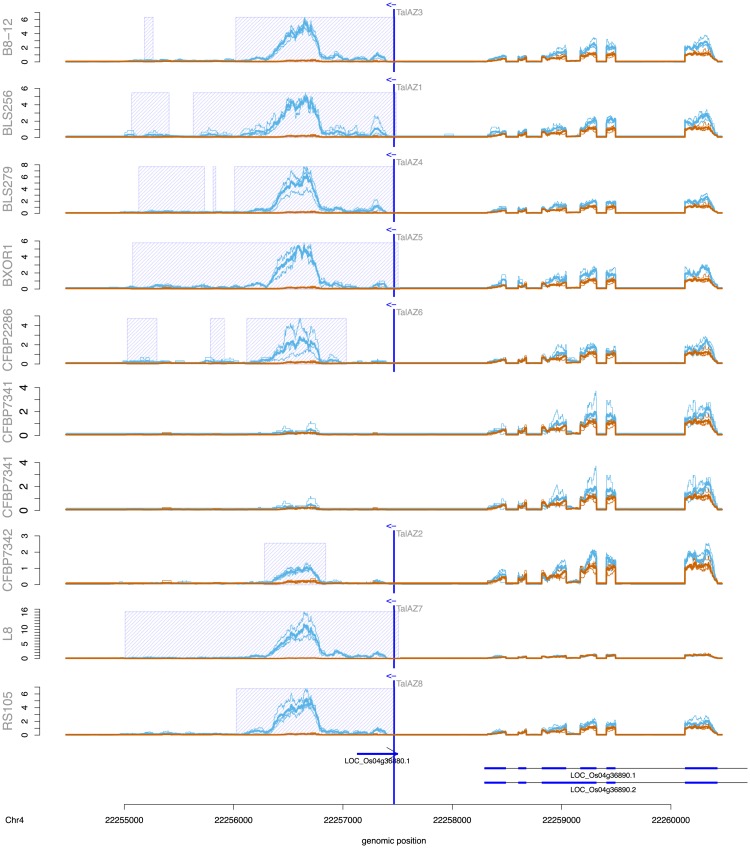
Genome-wide predictions of TalAZ in *Oryza sativa* Nipponbare profile for 10 *Xoc* strains in the area of the TalAZ target box. RNA-seq coverage after inoculation (blue line) is compared with mock control (brown line). In addition, we show the average of individual replicates of control and treatment are summarized as thick lines. The blue shaded boxes mark the differentially expressed regions. The arrows under the profiles reflect the MSU7 annotation within the genomic region. The genomic position of the TALE target box is marked by a vertical blue line.

In the following, we will discuss two example regions in detail. As discussed in the previous section, TalAZ appears to be an orphan TALE based on the promoterome-wide scans for target boxes. However, based on genome-wide scans, we find a differentially expressed region, which could constitute a target gene of TalAZ, on Chr4 (Fig 6). Only 8 of the 10 *Xoc* strains studied have a TalAZ member in their TALEome. The profile plots clearly show that the region of interest is only differentially expressed after infection with these 8 strains harbouring TalAZ members. Performing blast searches of the differentially expressed sequences, we received a hit for XP_015634381.1, a sulfated surface glycoprotein 185 [Oryza sativa Japonica Group], which has been added to the IRGSP-1.0 annotation at NCBI but was not present in MSU7.

**Fig 7:**
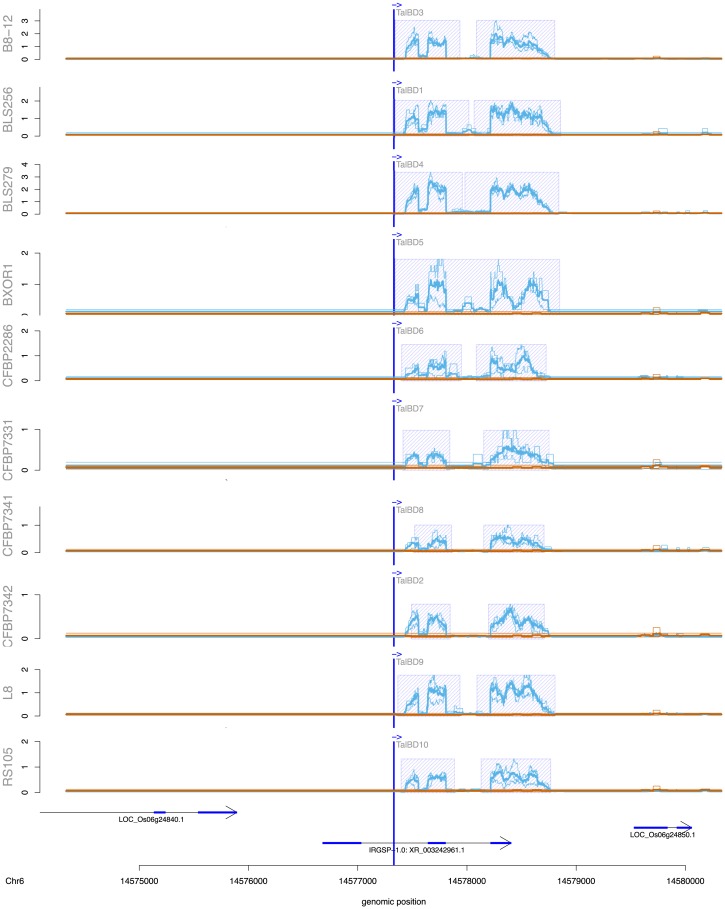
Genome-wide predictions of TalBD in *Oryza sativa* Nipponbare profile for 10 *Xoc* strains in the area of the TalBD target box. RNA-seq coverage after inoculation (blue line) is compared with mock control (brown line). In addition, we show the average of individual replicates of control and treatment are summarized as thick lines. The blue shaded boxes mark the differentially expressed regions. The arrows under the profiles reflect the MSU7 annotation within the genomic region. The genomic position of the TALE target box is marked by a vertical blue line.

As a second example, we consider a putative TalBD target on Chr6. The profile plots (Fig 7) show differentially expressed regions in all 10 strains. However, a blastx search of the respective sequences, spanning two larger differentially expression regions, provides no clear result. Matches include an Auxin-responsive protein IAA22 (Q69TU6.1) and different bromodomain-containing factors (XP_006659043.1, XP_025882131.1 XP_015650662.1). As drops in the coverage profiles and split reads in the mapping indicate the existence of introns within the differentially expressed regions, we additionally compare the spliced sequence using blastn against the NCBI reference RNA sequences. The result contains a predicted non-conding RNA (XR_003242961.1) and different transcript variants of a predicted mRNA, coding for bromodomain-containing factors (XM_015840709.1, XM_015840708.1, XM_006658980.2, XM_026026346.1, XM_015795177.2, XM_015795176.2).

In summary, our results demonstrate that genome-wide prediction of target boxes using PrediTALE enables us to identify novel targets independently of existing gene annotations including previously missing non-coding RNAs.

### Conclusion

Accurate computational predictions of TALE target boxes are required for elucidating virulence targets of TALEs that support bacterial infection of host plants. In this paper, we present PrediTALE, a novel approach for predicting target boxes based on a TALE’s RVD sequence. Since the publication of all previous approaches [14, 16, 18], our understanding of mechanisms and principles of TALE targeting has increased substantially. Specifically, it has been shown that repeats of aberrant lengths may compensate for frame shifts in target boxes [6], that activation of gene expression by TALEs binding to the reverse strand is possible, but rare [13]. In addition, quantitative data about virtually all combinations of AAs at RVD positions have been collected [19, 21–25]. All these insights have been integrated into PrediTALE either as part of the model or as training data that are used to adapt model parameters. Here, we demonstrate that PrediTALE predicts TALE targets with improved accuracy compared with previous approaches, where ground truth is derived from in-house and public RNA-seq data after *Xoo* and *Xoc* infection. However, our results also confirm that any of the current computational approaches suffers from false positive predictions and, hence, experimental support of predicted targets is essential.

PrediTALE predicts several unique target genes, several of which are highly promising for further experimental validation. While RNA-seq data supports that these are activated by TALEs *in planta*, their importance for the infection process still needs to be investigated.

Previously, predictions have been mostly limited to putative promoter regions of annotated genes. Here, we consider genome-wide predictions instead, which are feasible due to the acceptable runtime of PrediTALE, the improved accuracy of target box predictions, and the filtering steps based on RNA-seq data as implemented in DerTALE. We demonstrate that targets reported from promoterome-wide predictions are also recovered in genome-wide scans, but we also find differentially expressed regions at loci that do not overlap with annotated genes. These could be either protein-coding genes that are missing from the current annotation, but also include putative non-coding RNAs, which might have regulatory activity or other functions that foster bacterial infection.

To promote future research in plant-pathogen interactions related to TALEs, we make our methods available to the scientific community as open-source software tools.

## Supporting information

S1 Text Preprocessing of training data. (PDF)

S2 Text Supplementary tables. Supplementary Tables A to T.
(PDF)

S1 Table Gene abundances and sleuth output for *Xoo* strains. (XLS)

S2 Table Gene abundances and sleuth output for *Xoc* strains. (XLS)

S3 Table Complete list of top 20 predictions for all approaches and *Xoo* and *Xoc* strains. (XLS)

S4 Table Results of genome-wide predictions for *Xoo* strains. (XLS)

S5 Table Results of genome-wide predictions for *Xoc* strains. (XLS)

S6 Table PrediTALE predictions of target boxes for TALEs from other *Xanthomonas* species with validated virulence targets in the corresponding host plant. (XLS)

S1 Data RVD sequences of all *Xoo* and *Xoc* TALEs considered in this manuscript. (FASTA)

S1 Fig Venn diagrams of predictions of the four approaches considered. For each *Xoc* strain and each approach, we consider the set of target genes obtained as the union of the top 20 predictions per TALE.
(PDF)

S2 Fig Venn diagrams of true positive predictions of the four approaches considered. For each *Xoc* strain and each approach, we consider the set of target genes obtained as the union of the top 20 predictions per TALE. These sets are filtered by up-regulation of the corresponding genes according to RNA-seq data, and the resulting subsets are displayed.
(PDF)

S3 Fig Performance evaluation on the level of target genes for three *Xoo* strains. For each approach, we plot the number of predicted target genes that are also up-regulated in the infection (true positives, TPs; q-value < 0.01, log fold change > 2) against the number of predicted target sites per TALE.
(PDF)

S4 Fig Performance evaluation on the level of TALEs for three *Xoo* strains. For each approach, we plot the number of TALEs with at least one predicted target gene that is also up-regulated in the infection (q-value < 0.01, log fold change > 2) against the number of predicted target sites per TALE.
(PDF)

S5 Fig Performance evaluation on the level of target genes for three *Xoo* strains when filtering for predictions of TALE boxes on the same strand as the downstream gene. For each approach, we plot the number of predicted target genes that are also up-regulated in the infection (true positives, TPs) against the number of predicted target sites per TALE.
(PDF)

S6 Fig Performance evaluation on the level of TALEs for three *Xoo* strains when filtering for predictions of TALE boxes on the same strand as the downstream gene. For each approach, we plot the number of TALEs with at least one predicted target gene that is also up-regulated in the infection against the number of predicted target sites per TALE.
(PDF)

S7 Fig Performance evaluation on the level of TALEs for 10 *Xoc* strains. For each approach, we plot the number of TALEs with at least one predicted target gene that is also up-regulated in the infection against the number of predicted target sites per TALE.
(PDF)

S8 Fig Performance evaluation on the level of target genes for 10 *Xoc* strains. For each approach, we plot the number of predicted target genes that are also up-regulated in the infection (true positives, TPs; q-value < 0.05, log fold change > 2) against the number of predicted target sites per TALE.
(PDF)

S9 Fig Performance evaluation on the level of TALEs for 10 *Xoc* strains. For each approach, we plot the number of TALEs with at least one predicted target gene that is also up-regulated in the infection (q-value < 0.05, log fold change > 2) against the number of predicted target sites per TALE.
(PDF)

S10 Fig Performance evaluation on the level of target genes for 10 *Xoc* strains. For each approach, we plot the number of predicted target genes that are also up-regulated in the infection (true positives, TPs; q-value < 0.01, log fold change > 1) against the number of predicted target sites per TALE.
(PDF)

S11 Fig Performance evaluation on the level of TALEs for 10 *Xoc* strains. For each approach, we plot the number of TALEs with at least one predicted target gene that is also up-regulated in the infection (q-value < 0.01, log fold change > 1) against the number of predicted target sites per TALE.
(PDF)

S12 Fig Performance evaluation on the level of target genes for 10 *Xoc* strains when filtering for predictions of TALE boxes on the same strand as the downstream gene. For each approach, we plot the number of predicted target genes that are also up-regulated in the infection (true positives, TPs) against the number of predicted target sites per TALE.
(PDF)

S13 Fig Performance evaluation on the level of TALEs for 10 *Xoc* strains when filtering for predictions of TALE boxes on the same strand as the downstream gene. For each approach, we plot the number of TALEs with at least one predicted target gene that is also up-regulated in the infection against the number of predicted target sites per TALE.
(PDF)

S14 Fig Comparing the full model to the baseline model using only specificities based on AA 13 of an RVD and independent parameters for position 0. For each subset of features, we additionally compare the case where i) features are completing the full model and ii) features are added to the baseline model. We show violin plots of the number of true positive target gene predictions using at most 20 predictions per TALE including individual points for all *Xoo* and *Xoc* data sets.
(PDF)

S15 Fig Comparative visualization of the parameters of four different models. These are the baseline model using only specificities based on AA 13 of an RVD and independent parameters for position 0, a baseline model with conditional parameters for position 0 added, the full model except conditional parameters for position 0, and the full PrediTALE model. We find substantial differences between the specificity parameters of the four models, also in the parameters for specificities based on AA 13 of an RVD, although these are included into all four models. By contrast, we do not find a major difference between the position distributions learned for the full model and the full model except conditional parameters for position 0.
(PDF)

S16 Fig Presence of TALE classes in the three *Xoo* strains studied according to AnnoTALE. (PDF)

S17 Fig Log fold changes of the genes that are present among the top 20 predicted target genes of any of the four approaches and that are up-regulated in at least one of the *Xoo* strains. (PDF)

S18 Fig Genome-wide prediction of TalAO16 in *Oryza sativa* Nipponbare with corresponding RNA-seq data. RNA-seq coverage after inoculation (blue line) is compared with mock control (brown line). In addition, we show the average of individual replicates of control and treatment are summarized as thick lines. The blue shaded boxes mark the differentially expressed regions. The arrows under the profiles reflect the MSU7 annotation within the genomic region. The genomic position of the TALE target box is marked by a vertical blue line.
(PDF)
