# Supplementary material for: PrediTALE: A novel model learned from quantitative data allows for new perspectives on TALE targeting
Source: PLoS Comput Biol. 2019 Jul 11;15(7):e1007206. doi: 10.1371/journal.pcbi.1007206 (PMC6650089; doi:10.1371/journal.pcbi.1007206)
Supplement: S1 Text — (PDF) [file pcbi.1007206.s001.pdf]

S1 Text – Preprocessing of training data

Cong et al.

Pairs of TALE RVD sequence and tested target boxes were obtained from Fig. 1 and Fig. 2a of [22]. Data were grouped by TALE, and the global weight was computed as the maximum "Normalized reporter activation" for the current TALE divided by the maximum "Normalized reporter activation" reported for all TALEs with the same 13th AA at the varied positions. Target values were computed as the "Normalized reporter activation" of the current pair of TALE and target box divided by maximum "Normalized reporter activation" over all tested target boxes for the current TALE.

Streubel et al.

Pairs of TALE RVD sequence and tested target boxes were obtained from [19]. Data were grouped by TALE, and the global weight was computed as the maximum GUS activity for the current TALE divided by the maximum GUS activity reported for all TALEs with the same 13th AA at the varied positions stemming from the same experiment. Target values were computed as the GUS activity of the current pair of TALE and target box divided by maximum GUS activity over all tested target boxes for the current TALE.

Schreiber et al.

Pairs of TALE RVD sequence and tested target boxes were obtained from [23]. Data were grouped by TALE, and the global weight was computed as the maximum GUS activity for the current TALE divided by the maximum GUS activity reported for all TALEs stemming from the same experiment (corresponding to the same (sub-)figure in [23]). Target values were computed as the GUS activity of the current pair of TALE and target box divided by maximum GUS activity over all tested target boxes for the current TALE.

Yang et al.

Pairs of TALE RVD sequence and tested target boxes from [24] were provided by Wensheng Wei. Data were grouped by TALE, and the global weight was computed as the maximum EGFP activity for the current TALE divided by the maximum EGFP activity reported for all TALEs with the same 13th AA at the varied positions. Target values were computed as the EGFP activity of the current pair of TALE and target box divided by maximum EGFP activity over all tested target boxes for the current TALE.

Miller et al.

Pairs of TALE RVD sequence and tested target boxes were obtained from Supplementary Table 2 of [25]. Data were grouped by TALE, and the global weight was computed as the maximum "Normalized ELISA score" for the current TALE divided by the maximum "Normalized ELISA score" reported for all TALEs with the same 13th AA at the varied

positions. Target values were computed as the "Normalized ELISA score" of the current pair of TALE and target box divided by maximum "Normalized ELISA score" over all tested target boxes for the current TALE.

Rogers et al.

Probe sequences and binding intensities were obtained from [21] for 21 TALEs measured at different concentrations yielding a total of 55 PBM experiments. Each PBM experiment is accompanied by the RVD sequence of the corresponding TALE, where the number of RVDs ranges from 9 to 19. These 55 experiments were further filtered by data quality measured for each experiment individually.

Specifically, for probe sequence i, a score profile was computed using the model of TALgetter [18] based on the respective TALE and the log-probability of the best-matching sub-sequence on either the forward or the reverse complement strand of the probe sequence was stored as s_i. In addition, the number of sub-sequences with a log-probability larger than $s_i - \log(10)$ (i.e., those with a probability that is at most 10-fold lower than that of the best-matching sub-sequence) was stored as n_i. Using these measures, the Pearson correlation coefficient ρ was computed based on scores and mean normalized log-intensities for each best-matching sub-sequence. To this end, the unique set of all best-matching sub-sequences m_j was constructed and for each m_j all probe sequences containing m_j as the best-matching sub-sequence were collected, i.e., the m_j define a partitioning on the probe sequences. For each partition belonging to one specific m_j, log-intensities I_i were collected, divided by the corresponding n_i and averaged over all probe sequences in the current partition, yielding mean log-intensity values $\bar{I}_{m_j}$. In addition, each m_j has also been assigned a score by the TALgetter model denoted as s_{m_j}. Pearson correlation ρ was finally computed between the $\bar{I}_{m_j}$ and s_{m_j} values. Only PBM data sets with $\rho > 0.6$ were retained.

In addition, probe sequences were partitioned into "positives" and "negatives" based on the PBM intensity values. Probe sequences with a log-intensity more than two standard deviations above the mean log-intensity of the current PBM experiment were assigned to the "positive" class (or the top 50 probe sequences if this rule yielded less than 50 sequences) and all remaining probe sequences were assigned to the "negative" class. Probe sequences were also scored by their log-probability according to the TALgetter model, i.e., by the log of the mean probability of all corresponding sub-sequences. These scores were then used as classification scores to compute the area under the precision-recall curve [38] (AUC-PR) for the given binary classification problem. Only PBM experiments with an AUC-PR above 0.5 were retained for further analyes.

Probe sequences and PBM intensities of those experiments meeting both selection criteria were further processed to yield the final training data. Specifically, the unique best-matching sub-sequences m_j and corresponding average log-intenties I_{m_j} were collected, and the target values were set to the average log-intenties I_{m_j} normalized to the maximum I_{m_j} per PBM experiment. Global weights were defined identically for all m_j from a common PBM experiment and set such that all PBM experiments yield the same total (i.e., summed) global weight and all PBM experiments together obtain a total

global weight of 200, i.e., to a total global weight that is similar to that of 200 groups from one of the other experiments.
