# Supplementary material for: PrediTALE: A novel model learned from quantitative data allows for new perspectives on TALE targeting
Source: PLoS Comput Biol. 2019 Jul 11;15(7):e1007206. doi: 10.1371/journal.pcbi.1007206 (PMC6650089; doi:10.1371/journal.pcbi.1007206)
Supplement: S2 Text — Supplementary Tables A to T. (PDF) [file pcbi.1007206.s002.pdf]

Supplementary Tables A to T

Strain	#DEGs ($q < 0.01$, $lfc > 2$)	#DEGs ($p < 0.05$, $lfc > \log(2)$)
ICMP3125	7	107
PXO142	2	43
PXO83	2	49

Table A. Number of differentially expressed genes (DEGs) using the specified thresholds on p/q-values and log fold changes (lfc), respectively, considering RNA-seq data for three *Xoo* strains compared with mock inoculation.

Strain	#DEGs
B8-12	628
BLS256	652
BLS279	567
BXOR1	443
CFBP2286	202
CFBP7331	368
CFBP7341	328
CFBP7342	494
L8	672
RS105	335

Table B. Number of differentially expressed genes (DEGs) using a threshold of 0.01 on the FDR-corrected q-values and a threshold of 2 on the log fold change considering RNA-seq data for ten *Xoc* strains compared with mock inoculation.

strain	# predictions	Target.Finder	TALgetter	Talvez	PrediTALE
ICMP3125	1	1 (4)	3 (2)	2 (3)	4 (1)
PXO142	1	3 (1)	3 (1)	1 (4)	2 (3)
PXO83	1	3 (1)	3 (1)	1 (4)	3 (1)
avg. rank	1	2.00	1.33	3.67	1.67
ICMP3125	10	6 (2)	5 (3)	5 (3)	7 (1)
PXO142	10	4 (3)	5 (2)	4 (3)	7 (1)
PXO83	10	3 (4)	4 (2)	4 (2)	5 (1)
avg. rank	10	3.00	2.33	2.67	1.00
ICMP3125	20	6 (4)	8 (2)	8 (2)	10 (1)
PXO142	20	5 (2)	5 (2)	5 (2)	8 (1)
PXO83	20	3 (4)	4 (2)	4 (2)	7 (1)
avg. rank	20	3.33	2.00	2.00	1.00
ICMP3125	50	10 (4)	12 (2)	11 (3)	14 (1)
PXO142	50	5 (3)	6 (2)	5 (3)	9 (1)
PXO83	50	5 (3)	6 (2)	5 (3)	7 (1)
avg. rank	50	3.33	2.00	3.00	1.00
ICMP3125	Genes AUC	355 (4)	393 (2)	361 (3)	501 (1)
PXO142	Genes AUC	232 (3)	247 (2)	232 (3)	391 (1)
PXO83	Genes AUC	199 (3)	214 (2)	199 (3)	320 (1)
avg. rank	Genes AUC	3.33	2.00	3.00	1.00

Table C. Performance evaluation on the level of target genes for three *Xoo* strains. For each strain and each approach, we list the number of predicted target genes that are also up-regulated in the infection for different thresholds on the number of predictions per TALE, i.e., prediction cutoffs. For each threshold, we additionally report the average rank of each tool.

strain	# predictions	Target.Finder	TALgetter	Talvez	PrediTALE
ICMP3125	1	1 (4)	3 (2)	2 (3)	4 (1)
PXO142	1	3 (1)	3 (1)	1 (4)	2 (3)
PXO83	1	3 (1)	3 (1)	1 (4)	3 (1)
avg. rank	1	2.00	1.33	3.67	1.67
ICMP3125	10	6 (1)	5 (3)	5 (3)	6 (1)
PXO142	10	4 (3)	5 (1)	4 (3)	5 (1)
PXO83	10	3 (4)	4 (2)	4 (2)	5 (1)
avg. rank	10	2.67	2.00	2.67	1.00
ICMP3125	20	6 (4)	7 (2)	7 (2)	9 (1)
PXO142	20	5 (2)	5 (2)	5 (2)	6 (1)
PXO83	20	3 (4)	4 (2)	4 (2)	7 (1)
avg. rank	20	3.33	2.00	2.00	1.00
ICMP3125	50	8 (3)	10 (1)	8 (3)	10 (1)
PXO142	50	5 (3)	6 (2)	5 (3)	7 (1)
PXO83	50	4 (3)	6 (2)	4 (3)	7 (1)
avg. rank	50	3.00	1.67	3.00	1.00
ICMP3125	TALEs AUC	328 (3)	355 (2)	321 (4)	418 (1)
PXO142	TALEs AUC	232 (3)	247 (2)	232 (3)	301 (1)
PXO83	TALEs AUC	179 (4)	214 (2)	197 (3)	320 (1)
avg. rank	TALEs AUC	3.33	2.00	3.33	1.00

Table D. Performance evaluation on the level of TALEs for three *Xoo* strains. For each strain and each approach, we list the number of TALEs with at least one predicted target gene that is also up-regulated in the infection for different thresholds on the number of predictions per TALE, i.e., prediction cutoffs. For each threshold, we additionally report the average rank of each tool.

strain	# predictions	Target.Finder	TALgetter	Talvez	PrediTALE
ICMP3125	1	1 (1)	1 (1)	1 (1)	1 (1)
PXO142	1	1 (1)	0 (2)	0 (2)	0 (2)
PXO83	1	1 (1)	1 (1)	0 (3)	0 (3)
avg. rank	1	1.00	1.33	2.00	2.00
ICMP3125	10	3 (1)	2 (4)	3 (1)	3 (1)
PXO142	10	1 (2)	1 (2)	1 (2)	2 (1)
PXO83	10	1 (1)	1 (1)	1 (1)	1 (1)
avg. rank	10	1.33	2.33	1.33	1.00
ICMP3125	20	3 (2)	3 (2)	3 (2)	4 (1)
PXO142	20	1 (2)	1 (2)	1 (2)	2 (1)
PXO83	20	1 (1)	1 (1)	1 (1)	1 (1)
avg. rank	20	1.67	1.67	1.67	1.00
ICMP3125	50	5 (1)	3 (4)	4 (2)	4 (2)
PXO142	50	1 (2)	1 (2)	1 (2)	2 (1)
PXO83	50	1 (1)	1 (1)	1 (1)	1 (1)
avg. rank	50	1.33	2.33	1.67	1.33
ICMP3125	Genes AUC	191 (1)	132 (4)	142 (3)	181 (2)
PXO142	Genes AUC	50 (2)	46 (4)	49 (3)	91 (1)
PXO83	Genes AUC	50 (1)	50 (1)	49 (3)	46 (4)
avg. rank	Genes AUC	1.33	3.00	3.00	2.33

Table E. Performance evaluation on the level of target genes for three *Xoo* strains. For each strain and each approach, we list the number of predicted target genes that are also up-regulated in the infection (q-value < 0.01, log fold change > 2) for different thresholds on the number of predictions per TALE, i.e., prediction cutoffs. For each threshold, we additionally report the average rank of each tool.

strain	# predictions	Target.Finder	TALgetter	Talvez	PrediTALE
ICMP3125	1	1 (1)	1 (1)	1 (1)	1 (1)
PXO142	1	1 (1)	0 (2)	0 (2)	0 (2)
PXO83	1	1 (1)	1 (1)	0 (3)	0 (3)
avg. rank	1	1.00	1.33	2.00	2.00
ICMP3125	10	3 (1)	2 (4)	3 (1)	3 (1)
PXO142	10	1 (1)	1 (1)	1 (1)	1 (1)
PXO83	10	1 (1)	1 (1)	1 (1)	1 (1)
avg. rank	10	1.00	2.00	1.00	1.00
ICMP3125	20	3 (2)	3 (2)	3 (2)	4 (1)
PXO142	20	1 (1)	1 (1)	1 (1)	1 (1)
PXO83	20	1 (1)	1 (1)	1 (1)	1 (1)
avg. rank	20	1.33	1.33	1.33	1.00
ICMP3125	50	4 (1)	3 (3)	3 (3)	4 (1)
PXO142	50	1 (1)	1 (1)	1 (1)	1 (1)
PXO83	50	1 (1)	1 (1)	1 (1)	1 (1)
avg. rank	50	1.00	1.67	1.67	1.00
ICMP3125	TALEs AUC	171 (2)	132 (4)	140 (3)	181 (1)
PXO142	TALEs AUC	50 (1)	46 (4)	49 (2)	48 (3)
PXO83	TALEs AUC	50 (1)	50 (1)	49 (3)	46 (4)
avg. rank	TALEs AUC	1.33	3.00	2.67	2.67

Table F. Performance evaluation on the level of TALEs for three *Xoo* strains. For each strain and each approach, we list the number of TALEs with at least one predicted target gene that is also up-regulated in the infection (q-value < 0.01, log fold change > 2) for different thresholds on the number of predictions per TALE, i.e., prediction cutoffs. For each threshold, we additionally report the average rank of each tool.

strain	# predictions	Target.Finder	TALgetter	Talvez	PrediTALE
ICMP3125	1	1 (4)	3 (2)	3 (2)	4 (1)
PXO142	1	3 (1)	3 (1)	1 (4)	2 (3)
PXO83	1	3 (1)	3 (1)	2 (4)	3 (1)
avg. rank	1	2.00	1.33	3.33	1.67
ICMP3125	10	6 (3)	6 (3)	8 (2)	9 (1)
PXO142	10	5 (2)	5 (2)	5 (2)	8 (1)
PXO83	10	3 (4)	4 (2)	4 (2)	7 (1)
avg. rank	10	3.00	2.33	2.00	1.00
ICMP3125	20	8 (2)	8 (2)	8 (2)	11 (1)
PXO142	20	5 (2)	5 (2)	5 (2)	8 (1)
PXO83	20	5 (2)	5 (2)	4 (4)	7 (1)
avg. rank	20	2.00	2.00	2.67	1.00
ICMP3125	50	10 (3)	15 (1)	10 (3)	14 (2)
PXO142	50	5 (3)	6 (2)	5 (3)	9 (1)
PXO83	50	6 (3)	10 (1)	6 (3)	7 (2)
avg. rank	50	3.00	1.33	3.00	1.67
ICMP3125	Genes AUC	387 (4)	490 (2)	412 (3)	563 (1)
PXO142	Genes AUC	242 (3)	266 (2)	240 (4)	402 (1)
PXO83	Genes AUC	226 (4)	302 (2)	241 (3)	328 (1)
avg. rank	Genes AUC	3.67	2.00	3.33	1.00

Table G. Performance evaluation on the level of target genes for three *Xoo* strains when filtering for predictions of TALE boxes on the same strand as the downstream gene. For each strain and each approach, we list the number of predicted target genes that are also up-regulated in the infection for different thresholds on the number of predictions per TALE, i.e., prediction cutoffs. For each threshold, we additionally report the average rank of each tool.

strain	# predictions	Target.Finder	TALgetter	Talvez	PrediTALE
ICMP3125	1	1 (4)	3 (2)	3 (2)	4 (1)
PXO142	1	3 (1)	3 (1)	1 (4)	2 (3)
PXO83	1	3 (1)	3 (1)	2 (4)	3 (1)
avg. rank	1	2.00	1.33	3.33	1.67
ICMP3125	10	6 (3)	6 (3)	7 (2)	8 (1)
PXO142	10	5 (2)	5 (2)	5 (2)	6 (1)
PXO83	10	3 (4)	4 (2)	4 (2)	7 (1)
avg. rank	10	3.00	2.33	2.00	1.00
ICMP3125	20	7 (2)	7 (2)	7 (2)	9 (1)
PXO142	20	5 (2)	5 (2)	5 (2)	6 (1)
PXO83	20	4 (3)	5 (2)	4 (3)	7 (1)
avg. rank	20	2.33	2.00	2.33	1.00
ICMP3125	50	7 (3)	10 (1)	7 (3)	10 (1)
PXO142	50	5 (3)	6 (2)	5 (3)	7 (1)
PXO83	50	5 (3)	8 (1)	5 (3)	7 (2)
avg. rank	50	3.00	1.33	3.00	1.33
ICMP3125	TALEs AUC	325 (3)	389 (2)	323 (4)	440 (1)
PXO142	TALEs AUC	242 (3)	266 (2)	240 (4)	312 (1)
PXO83	TALEs AUC	190 (4)	271 (2)	217 (3)	328 (1)
avg. rank	TALEs AUC	3.33	2.00	3.67	1.00

Table H. Performance evaluation on the level of TALEs for three *Xoo* strains when filtering for predictions of TALE boxes on the same strand as the downstream gene. For each strain and each approach, we list the number of TALEs with at least one predicted target gene that is also up-regulated in the infection for different thresholds on the number of predictions per TALE, i.e., prediction cutoffs. For each threshold, we additionally report the average rank of each tool.

strain	# predictions	Target.Finder	TALgetter	Talvez	PrediTALE
B8-12	1	6 (2)	5 (3)	4 (4)	7 (1)
BLS256	1	5 (2)	5 (2)	5 (2)	7 (1)
BLS279	1	4 (2)	3 (4)	4 (2)	5 (1)
BXOR1	1	4 (2)	2 (4)	5 (1)	3 (3)
CFBP2286	1	4 (2)	4 (2)	4 (2)	5 (1)
CFBP7331	1	4 (1)	2 (4)	3 (2)	3 (2)
CFBP7341	1	4 (1)	2 (3)	3 (2)	2 (3)
CFBP7342	1	5 (2)	2 (4)	4 (3)	6 (1)
L8	1	9 (2)	8 (3)	6 (4)	12 (1)
RS105	1	3 (2)	3 (2)	3 (2)	4 (1)
avg. rank	1	1.8	3.1	2.4	1.5
B8-12	10	16 (4)	19 (2)	22 (1)	18 (3)
BLS256	10	15 (4)	16 (3)	24 (1)	20 (2)
BLS279	10	12 (4)	15 (3)	17 (1)	17 (1)
BXOR1	10	17 (3)	15 (4)	19 (1)	18 (2)
CFBP2286	10	11 (3)	11 (3)	13 (1)	12 (2)
CFBP7331	10	10 (4)	12 (3)	14 (2)	15 (1)
CFBP7341	10	10 (4)	11 (2)	11 (2)	14 (1)
CFBP7342	10	11 (2)	11 (2)	10 (4)	13 (1)
L8	10	18 (4)	24 (2)	24 (2)	28 (1)
RS105	10	12 (4)	14 (1)	14 (1)	14 (1)
avg. rank	10	3.6	2.5	1.6	1.5
B8-12	20	23 (4)	27 (2)	25 (3)	29 (1)
BLS256	20	22 (4)	26 (2)	26 (2)	28 (1)
BLS279	20	19 (4)	22 (2)	21 (3)	27 (1)
BXOR1	20	18 (4)	20 (3)	23 (2)	26 (1)
CFBP2286	20	13 (4)	17 (2)	14 (3)	18 (1)
CFBP7331	20	15 (3)	15 (3)	16 (2)	19 (1)
CFBP7341	20	14 (2)	13 (3)	12 (4)	16 (1)
CFBP7342	20	16 (1)	16 (1)	13 (4)	14 (3)
L8	20	29 (4)	35 (2)	30 (3)	39 (1)
RS105	20	17 (3)	21 (1)	17 (3)	20 (2)
avg. rank	20	3.3	2.1	2.9	1.3
B8-12	50	45 (1)	41 (3)	40 (4)	45 (1)
BLS256	50	41 (3)	40 (4)	43 (2)	52 (1)
BLS279	50	36 (2)	33 (3)	33 (3)	47 (1)
BXOR1	50	33 (2)	31 (4)	33 (2)	36 (1)
CFBP2286	50	19 (4)	20 (3)	23 (2)	25 (1)
CFBP7331	50	24 (2)	24 (2)	21 (4)	26 (1)
CFBP7341	50	22 (2)	22 (2)	18 (4)	23 (1)
CFBP7342	50	26 (2)	25 (3)	24 (4)	27 (1)
L8	50	51 (3)	53 (2)	48 (4)	59 (1)
RS105	50	29 (2)	26 (4)	30 (1)	29 (2)
avg. rank	50	2.3	3.0	3.0	1.1
B8-12	Genes AUC	1331 (3)	1411 (2)	1297 (4)	1563 (1)
BLS256	Genes AUC	1210 (4)	1345 (3)	1446 (2)	1587 (1)
BLS279	Genes AUC	1018 (4)	1127 (2)	1102 (3)	1529 (1)
BXOR1	Genes AUC	1129 (3)	1072 (4)	1203 (2)	1331 (1)
CFBP2286	Genes AUC	655 (4)	759 (3)	809 (2)	913 (1)
CFBP7331	Genes AUC	789 (2)	770 (4)	788 (3)	998 (1)
CFBP7341	Genes AUC	762 (2)	699 (3)	643 (4)	877 (1)
CFBP7342	Genes AUC	877 (1)	839 (3)	742 (4)	860 (2)
L8	Genes AUC	1568 (4)	1859 (2)	1631 (3)	2125 (1)
RS105	Genes AUC	864 (4)	969 (2)	920 (3)	1047 (1)
avg. rank	Genes AUC	3.1	2.8	3.0	1.1

Table I. Performance evaluation on the level of target genes for ten *Xoc* strains. For each strain and each approach, we list the number of predicted target genes that are also up-regulated in the infection for different cutoffs on the number of predictions per TALE, i.e., prediction ranks. For each threshold, we additionally report the average rank of each tool.

strain	# predictions	Target.Finder	TALgetter	Talvez	PrediTALE
B8-12	1	6 (2)	5 (3)	4 (4)	7 (1)
BLS256	1	5 (2)	5 (2)	5 (2)	7 (1)
BLS279	1	4 (2)	3 (4)	4 (2)	5 (1)
BXOR1	1	4 (2)	2 (4)	5 (1)	3 (3)
CFBP2286	1	4 (2)	4 (2)	4 (2)	5 (1)
CFBP7331	1	4 (1)	2 (4)	3 (2)	3 (2)
CFBP7341	1	4 (1)	2 (3)	3 (2)	2 (3)
CFBP7342	1	5 (2)	2 (4)	4 (3)	6 (1)
L8	1	9 (2)	8 (3)	6 (4)	12 (1)
RS105	1	3 (2)	3 (2)	3 (2)	4 (1)
avg. rank	1	1.8	3.1	2.4	1.5
B8-12	10	11 (4)	13 (2)	16 (1)	13 (2)
BLS256	10	10 (4)	11 (3)	17 (1)	15 (2)
BLS279	10	8 (4)	10 (3)	12 (1)	12 (1)
BXOR1	10	13 (3)	11 (4)	15 (1)	14 (2)
CFBP2286	10	9 (4)	10 (2)	12 (1)	10 (2)
CFBP7331	10	7 (4)	9 (1)	9 (1)	9 (1)
CFBP7341	10	7 (3)	9 (1)	7 (3)	9 (1)
CFBP7342	10	10 (1)	10 (1)	9 (4)	10 (1)
L8	10	14 (4)	18 (2)	18 (2)	20 (1)
RS105	10	8 (4)	10 (2)	11 (1)	10 (2)
avg. rank	10	3.5	2.1	1.6	1.5
B8-12	20	15 (4)	19 (1)	17 (3)	19 (1)
BLS256	20	15 (4)	18 (2)	17 (3)	19 (1)
BLS279	20	12 (4)	15 (2)	14 (3)	19 (1)
BXOR1	20	14 (3)	14 (3)	17 (2)	18 (1)
CFBP2286	20	10 (4)	13 (1)	12 (3)	13 (1)
CFBP7331	20	9 (4)	10 (2)	10 (2)	11 (1)
CFBP7341	20	8 (3)	9 (2)	8 (3)	10 (1)
CFBP7342	20	11 (1)	10 (3)	11 (1)	10 (3)
L8	20	18 (4)	24 (1)	20 (3)	24 (1)
RS105	20	11 (4)	15 (1)	12 (3)	15 (1)
avg. rank	20	3.5	1.8	2.6	1.2
B8-12	50	24 (1)	22 (3)	20 (4)	23 (2)
BLS256	50	21 (2)	20 (4)	21 (2)	24 (1)
BLS279	50	20 (2)	18 (3)	17 (4)	24 (1)
BXOR1	50	19 (4)	21 (1)	20 (2)	20 (2)
CFBP2286	50	12 (4)	13 (3)	16 (1)	15 (2)
CFBP7331	50	12 (3)	13 (2)	10 (4)	14 (1)
CFBP7341	50	11 (3)	13 (2)	9 (4)	14 (1)
CFBP7342	50	13 (2)	13 (2)	14 (1)	13 (2)
L8	50	24 (3)	26 (2)	23 (4)	28 (1)
RS105	50	17 (2)	16 (4)	17 (2)	18 (1)
avg. rank	50	2.6	2.6	2.8	1.4
B8-12	TALEs AUC	862 (3)	878 (2)	822 (4)	923 (1)
BLS256	TALEs AUC	772 (4)	825 (3)	878 (2)	965 (1)
BLS279	TALEs AUC	664 (4)	704 (2)	704 (2)	937 (1)
BXOR1	TALEs AUC	759 (3)	749 (4)	830 (2)	840 (1)
CFBP2286	TALEs AUC	507 (4)	575 (3)	612 (2)	623 (1)
CFBP7331	TALEs AUC	447 (4)	502 (2)	457 (3)	561 (1)
CFBP7341	TALEs AUC	427 (3)	498 (2)	386 (4)	544 (1)
CFBP7342	TALEs AUC	545 (1)	522 (4)	534 (3)	543 (2)
L8	TALEs AUC	960 (4)	1115 (2)	978 (3)	1188 (1)
RS105	TALEs AUC	575 (4)	665 (2)	600 (3)	717 (1)
avg. rank	TALEs AUC	3.4	2.6	2.8	1.1

Table J. Performance evaluation on the level of TALEs for ten *Xoo* strains. For each strain and each approach, we list the number of TALEs with at least one predicted target gene that is also up-regulated in the infection for different cutoffs on the number of predictions per TALE, i.e., prediction ranks. For each threshold, we additionally report the average rank of each tool.

strain	# predictions	Target.Finder	TALgetter	Talvez	PrediTALE
B8-12	1	6 (2)	5 (3)	4 (4)	7 (1)
BLS256	1	5 (2)	5 (2)	5 (2)	7 (1)
BLS279	1	4 (2)	3 (4)	4 (2)	5 (1)
BXOR1	1	4 (2)	2 (4)	5 (1)	3 (3)
CFBP2286	1	4 (2)	4 (2)	4 (2)	5 (1)
CFBP7331	1	4 (1)	2 (4)	3 (2)	3 (2)
CFBP7341	1	4 (1)	2 (3)	3 (2)	2 (3)
CFBP7342	1	5 (2)	2 (4)	4 (3)	6 (1)
L8	1	9 (2)	8 (3)	6 (4)	12 (1)
RS105	1	3 (2)	3 (2)	3 (2)	4 (1)
avg. rank	1	1.8	3.1	2.4	1.5
B8-12	10	16 (4)	19 (2)	23 (1)	18 (3)
BLS256	10	15 (4)	16 (3)	24 (1)	20 (2)
BLS279	10	12 (4)	15 (3)	17 (1)	17 (1)
BXOR1	10	17 (3)	15 (4)	19 (1)	18 (2)
CFBP2286	10	11 (3)	11 (3)	13 (1)	12 (2)
CFBP7331	10	10 (4)	12 (3)	14 (2)	15 (1)
CFBP7341	10	10 (4)	11 (2)	11 (2)	14 (1)
CFBP7342	10	11 (2)	11 (2)	10 (4)	13 (1)
L8	10	18 (4)	24 (2)	24 (2)	28 (1)
RS105	10	12 (4)	14 (2)	15 (1)	14 (2)
avg. rank	10	3.6	2.6	1.6	1.6
B8-12	20	23 (4)	27 (2)	26 (3)	29 (1)
BLS256	20	22 (4)	26 (2)	26 (2)	28 (1)
BLS279	20	19 (4)	22 (2)	21 (3)	27 (1)
BXOR1	20	18 (4)	20 (3)	23 (2)	27 (1)
CFBP2286	20	13 (4)	17 (2)	14 (3)	18 (1)
CFBP7331	20	15 (3)	15 (3)	16 (2)	19 (1)
CFBP7341	20	14 (2)	13 (3)	12 (4)	16 (1)
CFBP7342	20	17 (1)	16 (2)	13 (4)	14 (3)
L8	20	29 (4)	35 (2)	30 (3)	39 (1)
RS105	20	17 (4)	21 (1)	18 (3)	20 (2)
avg. rank	20	3.4	2.2	2.9	1.3
B8-12	50	45 (1)	41 (3)	41 (3)	45 (1)
BLS256	50	41 (3)	41 (3)	43 (2)	52 (1)
BLS279	50	37 (2)	33 (4)	34 (3)	47 (1)
BXOR1	50	35 (3)	31 (4)	36 (2)	38 (1)
CFBP2286	50	20 (4)	21 (3)	23 (2)	26 (1)
CFBP7331	50	25 (2)	24 (3)	22 (4)	26 (1)
CFBP7341	50	22 (2)	22 (2)	18 (4)	23 (1)
CFBP7342	50	27 (1)	25 (3)	24 (4)	27 (1)
L8	50	52 (3)	53 (2)	49 (4)	59 (1)
RS105	50	30 (2)	26 (4)	31 (1)	30 (2)
avg. rank	50	2.3	3.1	2.9	1.1
B8-12	Genes AUC	1331 (4)	1411 (2)	1342 (3)	1563 (1)
BLS256	Genes AUC	1210 (4)	1346 (3)	1446 (2)	1587 (1)
BLS279	Genes AUC	1046 (4)	1127 (3)	1130 (2)	1529 (1)
BXOR1	Genes AUC	1163 (3)	1072 (4)	1242 (2)	1395 (1)
CFBP2286	Genes AUC	668 (4)	782 (3)	809 (2)	921 (1)
CFBP7331	Genes AUC	803 (3)	770 (4)	817 (2)	998 (1)
CFBP7341	Genes AUC	762 (2)	699 (3)	643 (4)	877 (1)
CFBP7342	Genes AUC	911 (1)	839 (3)	742 (4)	860 (2)
L8	Genes AUC	1596 (4)	1859 (2)	1659 (3)	2125 (1)
RS105	Genes AUC	892 (4)	969 (2)	965 (3)	1063 (1)
avg. rank	Genes AUC	3.3	2.9	2.7	1.1

Table K. Performance evaluation on the level of target genes for ten *Xoc* strains. For each strain and each approach, we list the number of predicted target genes that are also up-regulated in the infection (q-value < 0.05, log fold change > 2) for different cutoffs on the number of predictions per TALE, i.e., prediction ranks. For each threshold, we additionally report the average rank of each tool.

strain	# predictions	Target.Finder	TALgetter	Talvez	PrediTALE
B8-12	1	6 (2)	5 (3)	4 (4)	7 (1)
BLS256	1	5 (2)	5 (2)	5 (2)	7 (1)
BLS279	1	4 (2)	3 (4)	4 (2)	5 (1)
BXOR1	1	4 (2)	2 (4)	5 (1)	3 (3)
CFBP2286	1	4 (2)	4 (2)	4 (2)	5 (1)
CFBP7331	1	4 (1)	2 (4)	3 (2)	3 (2)
CFBP7341	1	4 (1)	2 (3)	3 (2)	2 (3)
CFBP7342	1	5 (2)	2 (4)	4 (3)	6 (1)
L8	1	9 (2)	8 (3)	6 (4)	12 (1)
RS105	1	3 (2)	3 (2)	3 (2)	4 (1)
avg. rank	1	1.8	3.1	2.4	1.5
B8-12	10	11 (4)	13 (2)	17 (1)	13 (2)
BLS256	10	10 (4)	11 (3)	17 (1)	15 (2)
BLS279	10	8 (4)	10 (3)	12 (1)	12 (1)
BXOR1	10	13 (3)	11 (4)	15 (1)	14 (2)
CFBP2286	10	9 (4)	10 (2)	12 (1)	10 (2)
CFBP7331	10	7 (4)	9 (1)	9 (1)	9 (1)
CFBP7341	10	7 (3)	9 (1)	7 (3)	9 (1)
CFBP7342	10	10 (1)	10 (1)	9 (4)	10 (1)
L8	10	14 (4)	18 (2)	18 (2)	20 (1)
RS105	10	8 (4)	10 (2)	12 (1)	10 (2)
avg. rank	10	3.5	2.1	1.6	1.5
B8-12	20	15 (4)	19 (1)	18 (3)	19 (1)
BLS256	20	15 (4)	18 (2)	17 (3)	19 (1)
BLS279	20	12 (4)	15 (2)	14 (3)	19 (1)
BXOR1	20	14 (3)	14 (3)	17 (2)	18 (1)
CFBP2286	20	10 (4)	13 (1)	12 (3)	13 (1)
CFBP7331	20	9 (4)	10 (2)	10 (2)	11 (1)
CFBP7341	20	8 (3)	9 (2)	8 (3)	10 (1)
CFBP7342	20	11 (1)	10 (3)	11 (1)	10 (3)
L8	20	18 (4)	24 (1)	20 (3)	24 (1)
RS105	20	11 (4)	15 (1)	13 (3)	15 (1)
avg. rank	20	3.5	1.8	2.6	1.2
B8-12	50	24 (1)	22 (3)	20 (4)	23 (2)
BLS256	50	21 (2)	20 (4)	21 (2)	24 (1)
BLS279	50	21 (2)	18 (3)	18 (3)	24 (1)
BXOR1	50	19 (4)	21 (1)	20 (2)	20 (2)
CFBP2286	50	13 (4)	14 (3)	16 (1)	16 (1)
CFBP7331	50	13 (2)	13 (2)	11 (4)	14 (1)
CFBP7341	50	11 (3)	13 (2)	9 (4)	14 (1)
CFBP7342	50	13 (2)	13 (2)	14 (1)	13 (2)
L8	50	25 (3)	26 (2)	24 (4)	28 (1)
RS105	50	18 (1)	16 (4)	17 (3)	18 (1)
avg. rank	50	2.4	2.6	2.8	1.3
B8-12	TALEs AUC	862 (4)	878 (2)	864 (3)	923 (1)
BLS256	TALEs AUC	772 (4)	825 (3)	878 (2)	965 (1)
BLS279	TALEs AUC	692 (4)	704 (3)	732 (2)	937 (1)
BXOR1	TALEs AUC	759 (3)	749 (4)	830 (2)	840 (1)
CFBP2286	TALEs AUC	520 (4)	598 (3)	612 (2)	631 (1)
CFBP7331	TALEs AUC	461 (4)	502 (2)	486 (3)	561 (1)
CFBP7341	TALEs AUC	427 (3)	498 (2)	386 (4)	544 (1)
CFBP7342	TALEs AUC	545 (1)	522 (4)	534 (3)	543 (2)
L8	TALEs AUC	988 (4)	1115 (2)	1006 (3)	1188 (1)
RS105	TALEs AUC	603 (4)	665 (2)	642 (3)	717 (1)
avg. rank	TALEs AUC	3.5	2.7	2.7	1.1

Table L. Performance evaluation on the level of TALEs for ten *Xoo* strains. For each strain and each approach, we list the number of TALEs with at least one predicted target gene that is also up-regulated in the infection (q-value < 0.05, log fold change > 2) for different cutoffs on the number of predictions per TALE, i.e., prediction ranks. For each threshold, we additionally report the average rank of each tool.

measure	Target Finder	TALgetter	Talvez	PrediTALE	Quade	TALgetter vs TALESF	Talvez vs Target Finder	Talvez vs TALgetter	PrediTALE vs Target Finder	PrediTALE vs TALgetter	PrediTALE vs Talvez
TALEs R1	1.8	3.1	2.4	1.5	**	—	-			+++	++
TALEs R10	3.5	2.1	1.6	1.5	***	+	+++	++	+++		
TALEs R20	3.5	1.8	2.6	1.2	***	+++	+	-	+++		+++
TALEs R50	2.4	2.6	2.8	1.3	**				++	+++	+++
TALEs AUC	3.5	2.7	2.7	1.1	***	++	++		+++	+++	+++
Genes R1	1.8	3.1	2.4	1.5	**	—	-			+++	++
Genes R10	3.6	2.6	1.6	1.6	***	+	+++	+	+++	+	
Genes R20	3.4	2.2	2.9	1.3	***	+++	+		+++	++	+++
Genes R50	2.3	3.1	2.9	1.1	**				+++	+++	+++
Genes AUC	3.3	2.9	2.7	1.1	***		+		+++	+++	+++

Table M. Testing the significance of differences in prediction performance (q-value < 0.05, log fold change > 2). For each tool and each measure (TALEs/Genes; rank cutoff), we report the average rank per tool, the significance of the Quade test (*:< 0.05; **:< 0.01; ***:< 0.001), and the significance of the pairwise comparison in a post-hoc test. Here, '+' and '-' indicate that the first tool has gained a significantly better or worse performance than the second one, respectively. The number of symbols encodes the significance level in analogy to the Quade test.

strain	# predictions	Target.Finder	TALgetter	Talvez	PrediTALE
B8-12	1	8 (2)	8 (2)	5 (4)	11 (1)
BLS256	1	7 (3)	8 (2)	5 (4)	10 (1)
BLS279	1	6 (3)	7 (2)	5 (4)	8 (1)
BXOR1	1	5 (2)	5 (2)	6 (1)	4 (4)
CFBP2286	1	5 (3)	6 (2)	4 (4)	7 (1)
CFBP7331	1	6 (2)	7 (1)	5 (3)	5 (3)
CFBP7341	1	6 (1)	2 (3)	3 (2)	2 (3)
CFBP7342	1	5 (4)	7 (1)	6 (3)	7 (1)
L8	1	11 (2)	9 (3)	6 (4)	14 (1)
RS105	1	5 (3)	7 (2)	4 (4)	9 (1)
avg. rank	1	2.5	2.0	3.3	1.7
B8-12	10	22 (4)	26 (3)	33 (1)	30 (2)
BLS256	10	18 (4)	26 (3)	29 (1)	28 (2)
BLS279	10	18 (4)	27 (3)	28 (2)	31 (1)
BXOR1	10	23 (4)	28 (1)	28 (1)	28 (1)
CFBP2286	10	17 (3)	16 (4)	18 (2)	19 (1)
CFBP7331	10	14 (4)	21 (3)	23 (2)	24 (1)
CFBP7341	10	14 (2)	12 (4)	14 (2)	17 (1)
CFBP7342	10	14 (4)	18 (2)	18 (2)	22 (1)
L8	10	28 (4)	38 (2)	36 (3)	40 (1)
RS105	10	16 (4)	20 (3)	23 (2)	26 (1)
avg. rank	10	3.7	2.8	1.8	1.2
B8-12	20	36 (4)	42 (2)	40 (3)	48 (1)
BLS256	20	31 (4)	40 (2)	36 (3)	46 (1)
BLS279	20	32 (4)	41 (2)	38 (3)	45 (1)
BXOR1	20	36 (3)	35 (4)	38 (1)	37 (2)
CFBP2286	20	25 (3)	26 (2)	22 (4)	29 (1)
CFBP7331	20	27 (4)	30 (1)	30 (1)	30 (1)
CFBP7341	20	20 (2)	17 (3)	16 (4)	21 (1)
CFBP7342	20	24 (2)	24 (2)	23 (4)	28 (1)
L8	20	43 (4)	57 (2)	47 (3)	58 (1)
RS105	20	28 (3)	31 (2)	28 (3)	35 (1)
avg. rank	20	3.3	2.2	2.9	1.1
B8-12	50	75 (4)	78 (2)	78 (2)	84 (1)
BLS256	50	72 (4)	78 (3)	79 (2)	96 (1)
BLS279	50	67 (4)	71 (3)	72 (2)	85 (1)
BXOR1	50	59 (3)	56 (4)	64 (1)	63 (2)
CFBP2286	50	38 (2)	33 (4)	36 (3)	39 (1)
CFBP7331	50	50 (2)	46 (3)	45 (4)	51 (1)
CFBP7341	50	37 (1)	33 (3)	30 (4)	37 (1)
CFBP7342	50	47 (3)	48 (2)	42 (4)	52 (1)
L8	50	85 (4)	102 (2)	88 (3)	103 (1)
RS105	50	47 (4)	49 (3)	51 (2)	57 (1)
avg. rank	50	3.1	2.9	2.7	1.1
B8-12	Genes AUC	2123 (4)	2382 (2)	2293 (3)	2647 (1)
BLS256	Genes AUC	1865 (4)	2260 (2)	2211 (3)	2675 (1)
BLS279	Genes AUC	1829 (4)	2207 (2)	2176 (3)	2614 (1)
BXOR1	Genes AUC	1963 (3)	1909 (4)	2102 (2)	2125 (1)
CFBP2286	Genes AUC	1221 (4)	1240 (2)	1235 (3)	1453 (1)
CFBP7331	Genes AUC	1507 (4)	1537 (2)	1528 (3)	1708 (1)
CFBP7341	Genes AUC	1194 (2)	977 (3)	928 (4)	1217 (1)
CFBP7342	Genes AUC	1385 (3)	1438 (2)	1324 (4)	1665 (1)
L8	Genes AUC	2525 (4)	3182 (2)	2762 (3)	3339 (1)
RS105	Genes AUC	1398 (4)	1637 (2)	1571 (3)	1901 (1)
avg. rank	Genes AUC	3.6	2.3	3.1	1.0

Table N. Performance evaluation on the level of target genes for ten *Xoc* strains. For each strain and each approach, we list the number of predicted target genes that are also up-regulated in the infection (q-value < 0.01, log fold change > 1) for different cutoffs on the number of predictions per TALE, i.e., prediction ranks. For each threshold, we additionally report the average rank of each tool.

strain	# predictions	Target.Finder	TALgetter	Talvez	PrediTALE
B8-12	1	8 (2)	8 (2)	5 (4)	11 (1)
BLS256	1	7 (3)	8 (2)	5 (4)	10 (1)
BLS279	1	6 (3)	7 (2)	5 (4)	8 (1)
BXOR1	1	5 (2)	5 (2)	6 (1)	4 (4)
CFBP2286	1	5 (3)	6 (2)	4 (4)	7 (1)
CFBP7331	1	6 (2)	7 (1)	5 (3)	5 (3)
CFBP7341	1	6 (1)	2 (3)	3 (2)	2 (3)
CFBP7342	1	5 (4)	7 (1)	6 (3)	7 (1)
L8	1	11 (2)	9 (3)	6 (4)	14 (1)
RS105	1	5 (3)	7 (2)	4 (4)	9 (1)
avg. rank	1	2.5	2.0	3.3	1.7
B8-12	10	15 (4)	16 (3)	21 (1)	20 (2)
BLS256	10	13 (4)	16 (3)	19 (1)	19 (1)
BLS279	10	13 (4)	16 (3)	18 (2)	19 (1)
BXOR1	10	15 (4)	17 (3)	18 (1)	18 (1)
CFBP2286	10	13 (3)	13 (3)	15 (1)	15 (1)
CFBP7331	10	10 (4)	13 (2)	12 (3)	14 (1)
CFBP7341	10	9 (2)	9 (2)	9 (2)	10 (1)
CFBP7342	10	11 (3)	13 (2)	11 (3)	15 (1)
L8	10	17 (4)	23 (1)	20 (3)	22 (2)
RS105	10	12 (4)	13 (3)	17 (1)	17 (1)
avg. rank	10	3.6	2.5	1.8	1.2
B8-12	20	20 (4)	25 (1)	23 (2)	23 (2)
BLS256	20	18 (4)	22 (1)	20 (3)	22 (1)
BLS279	20	18 (4)	23 (1)	20 (3)	22 (2)
BXOR1	20	22 (1)	18 (4)	22 (1)	21 (3)
CFBP2286	20	15 (4)	17 (2)	17 (2)	18 (1)
CFBP7331	20	15 (2)	15 (2)	16 (1)	15 (2)
CFBP7341	20	10 (2)	10 (2)	10 (2)	11 (1)
CFBP7342	20	14 (2)	13 (3)	12 (4)	16 (1)
L8	20	21 (4)	28 (1)	23 (3)	26 (2)
RS105	20	17 (4)	20 (1)	18 (3)	20 (1)
avg. rank	20	3.1	1.8	2.4	1.6
B8-12	50	26 (4)	27 (2)	27 (2)	28 (1)
BLS256	50	26 (2)	26 (2)	26 (2)	27 (1)
BLS279	50	24 (3)	25 (2)	24 (3)	26 (1)
BXOR1	50	23 (2)	21 (4)	24 (1)	23 (2)
CFBP2286	50	19 (3)	17 (4)	20 (2)	21 (1)
CFBP7331	50	17 (3)	18 (2)	17 (3)	20 (1)
CFBP7341	50	13 (3)	15 (2)	13 (3)	18 (1)
CFBP7342	50	19 (3)	20 (1)	19 (3)	20 (1)
L8	50	28 (3)	29 (1)	27 (4)	29 (1)
RS105	50	19 (4)	21 (2)	21 (2)	23 (1)
avg. rank	50	3.0	2.2	2.5	1.1
B8-12	TALEs AUC	1031 (4)	1145 (2)	1118 (3)	1152 (1)
BLS256	TALEs AUC	971 (4)	1064 (2)	1022 (3)	1091 (1)
BLS279	TALEs AUC	954 (4)	1058 (2)	1008 (3)	1109 (1)
BXOR1	TALEs AUC	973 (3)	909 (4)	1023 (1)	1005 (2)
CFBP2286	TALEs AUC	735 (4)	766 (3)	819 (2)	899 (1)
CFBP7331	TALEs AUC	677 (4)	753 (2)	723 (3)	799 (1)
CFBP7341	TALEs AUC	498 (4)	559 (2)	500 (3)	613 (1)
CFBP7342	TALEs AUC	693 (3)	764 (2)	679 (4)	863 (1)
L8	TALEs AUC	1146 (4)	1302 (1)	1166 (3)	1269 (2)
RS105	TALEs AUC	795 (4)	896 (2)	870 (3)	974 (1)
avg. rank	TALEs AUC	3.8	2.2	2.8	1.2

Table O. Performance evaluation on the level of TALEs for ten *Xoo* strains. For each strain and each approach, we list the number of TALEs with at least one predicted target gene that is also up-regulated in the infection (q-value < 0.01, log fold change > 1) for different cutoffs on the number of predictions per TALE, i.e., prediction ranks. For each threshold, we additionally report the average rank of each tool.

measure	Target Finder	TALgetter	Talvez	PrediTALE	Quade	TALgetter vs TALESF	Talvez vs Target Finder	Talvez vs TALgetter	PrediTALE vs Target Finder	PrediTALE vs TALgetter	PrediTALE vs Talvez
TALEs R1	2.5	2	3.3	1.7	**		-	−	+	+	+++
TALEs R10	3.6	2.5	1.8	1.2	***	++	+++		+++	+	
TALEs R20	3.1	1.8	2.4	1.6	*	+++		-	+++		
TALEs R50	3	2.2	2.5	1.1	**				+++	++	+++
TALEs AUC	3.8	2.2	2.8	1.2	***	+++	+	-	+++	+	+++
Genes R1	2.5	2	3.3	1.7	**		-	−	+	+	+++
Genes R10	3.7	2.8	1.8	1.2	***	+	+++	+	+++	+++	
Genes R20	3.3	2.2	2.9	1.1	***	+++		-	+++	+	+++
Genes R50	3.1	2.9	2.7	1.1	***		+		+++	+++	+++
Genes AUC	3.6	2.3	3.1	1	***	+++		-	+++	++	+++

Table P. Testing the significance of differences in prediction performance (q-value < 0.01, log fold change > 1). For each tool and each measure (TALEs/Genes; rank cutoff), we report the average rank per tool, the significance of the Quade test (*:< 0.05; **:< 0.01; ***:< 0.001), and the significance of the pairwise comparison in a post-hoc test. Here, '+' and '-' indicate that the first tool has gained a significantly better or worse performance than the second one, respectively. The number of symbols encodes the significance level in analogy to the Quade test.

strain	# predictions	Target.Finder	TALgetter	Talvez	PrediTALE
B8-12	1	6 (2)	6 (2)	4 (4)	7 (1)
BLS256	1	5 (4)	6 (2)	6 (2)	7 (1)
BLS279	1	4 (2)	4 (2)	4 (2)	5 (1)
BXOR1	1	4 (2)	3 (3)	5 (1)	3 (3)
CFBP2286	1	4 (3)	5 (1)	4 (3)	5 (1)
CFBP7331	1	4 (1)	3 (2)	3 (2)	3 (2)
CFBP7341	1	4 (1)	2 (3)	3 (2)	2 (3)
CFBP7342	1	6 (1)	3 (4)	4 (3)	6 (1)
L8	1	9 (2)	9 (2)	7 (4)	11 (1)
RS105	1	3 (3)	4 (1)	3 (3)	4 (1)
avg. rank	1	2.1	2.2	2.6	1.5
B8-12	10	20 (4)	23 (1)	22 (3)	23 (1)
BLS256	10	18 (4)	22 (3)	24 (2)	25 (1)
BLS279	10	15 (4)	19 (2)	17 (3)	21 (1)
BXOR1	10	18 (3)	17 (4)	22 (2)	23 (1)
CFBP2286	10	12 (4)	13 (2)	13 (2)	14 (1)
CFBP7331	10	11 (4)	13 (2)	13 (2)	16 (1)
CFBP7341	10	10 (4)	12 (2)	11 (3)	15 (1)
CFBP7342	10	12 (1)	12 (1)	10 (4)	12 (1)
L8	10	22 (4)	29 (2)	27 (3)	31 (1)
RS105	10	14 (3)	17 (2)	14 (3)	18 (1)
avg. rank	10	3.5	2.1	2.7	1.0
B8-12	20	29 (4)	30 (2)	30 (2)	32 (1)
BLS256	20	26 (4)	30 (2)	33 (1)	30 (2)
BLS279	20	24 (3)	24 (3)	26 (2)	31 (1)
BXOR1	20	26 (2)	22 (4)	26 (2)	29 (1)
CFBP2286	20	16 (3)	16 (3)	19 (2)	20 (1)
CFBP7331	20	18 (2)	15 (4)	16 (3)	22 (1)
CFBP7341	20	18 (2)	14 (3)	14 (3)	19 (1)
CFBP7342	20	22 (1)	18 (2)	15 (4)	16 (3)
L8	20	35 (4)	40 (2)	37 (3)	44 (1)
RS105	20	20 (4)	21 (2)	21 (2)	23 (1)
avg. rank	20	2.9	2.7	2.4	1.3
B8-12	50	43 (4)	45 (3)	47 (1)	47 (1)
BLS256	50	42 (4)	44 (3)	53 (2)	56 (1)
BLS279	50	38 (3)	37 (4)	40 (2)	47 (1)
BXOR1	50	35 (4)	36 (3)	41 (2)	42 (1)
CFBP2286	50	22 (4)	23 (3)	25 (2)	26 (1)
CFBP7331	50	26 (3)	27 (2)	25 (4)	28 (1)
CFBP7341	50	25 (1)	24 (2)	21 (4)	24 (2)
CFBP7342	50	28 (3)	32 (1)	28 (3)	29 (2)
L8	50	50 (4)	54 (3)	58 (2)	63 (1)
RS105	50	28 (3)	27 (4)	35 (1)	29 (2)
avg. rank	50	3.3	2.8	2.3	1.3
B8-12	Genes AUC	1469 (4)	1626 (2)	1624 (3)	1685 (1)
BLS256	Genes AUC	1388 (4)	1544 (3)	1772 (2)	1799 (1)
BLS279	Genes AUC	1239 (4)	1342 (3)	1353 (2)	1675 (1)
BXOR1	Genes AUC	1249 (3)	1233 (4)	1417 (2)	1436 (1)
CFBP2286	Genes AUC	817 (4)	879 (3)	950 (2)	1008 (1)
CFBP7331	Genes AUC	871 (3)	891 (2)	846 (4)	1059 (1)
CFBP7341	Genes AUC	857 (2)	811 (3)	736 (4)	924 (1)
CFBP7342	Genes AUC	1008 (1)	970 (2)	859 (4)	913 (3)
L8	Genes AUC	1756 (4)	2033 (2)	2005 (3)	2255 (1)
RS105	Genes AUC	999 (4)	1083 (3)	1177 (1)	1140 (2)
avg. rank	Genes AUC	3.3	2.7	2.7	1.3

Table Q. Performance evaluation on the level of target genes for ten *Xoc* strains when filtering for predictions of TALE boxes on the same strand as the downstream gene. For each strain and each approach, we list the number of predicted target genes that are also up-regulated in the infection for different cutoffs on the number of predictions per TALE, i.e., prediction ranks. For each threshold, we additionally report the average rank of each tool.

strain	# predictions	Target.Finder	TALgetter	Talvez	PrediTALE
B8-12	1	6 (2)	6 (2)	4 (4)	7 (1)
BLS256	1	5 (4)	6 (2)	6 (2)	7 (1)
BLS279	1	4 (2)	4 (2)	4 (2)	5 (1)
BXOR1	1	4 (2)	3 (3)	5 (1)	3 (3)
CFBP2286	1	4 (3)	5 (1)	4 (3)	5 (1)
CFBP7331	1	4 (1)	3 (2)	3 (2)	3 (2)
CFBP7341	1	4 (1)	2 (3)	3 (2)	2 (3)
CFBP7342	1	6 (1)	3 (4)	4 (3)	6 (1)
L8	1	9 (2)	9 (2)	7 (4)	11 (1)
RS105	1	3 (3)	4 (1)	3 (3)	4 (1)
avg. rank	1	2.1	2.2	2.6	1.5
B8-12	10	14 (4)	16 (1)	16 (1)	16 (1)
BLS256	10	13 (4)	14 (3)	17 (1)	17 (1)
BLS279	10	11 (4)	13 (2)	12 (3)	15 (1)
BXOR1	10	13 (3)	13 (3)	16 (1)	16 (1)
CFBP2286	10	10 (4)	11 (2)	12 (1)	11 (2)
CFBP7331	10	7 (4)	9 (1)	8 (3)	9 (1)
CFBP7341	10	7 (3)	9 (1)	7 (3)	9 (1)
CFBP7342	10	10 (1)	9 (2)	9 (2)	9 (2)
L8	10	17 (4)	21 (1)	20 (3)	21 (1)
RS105	10	10 (4)	12 (2)	11 (3)	13 (1)
avg. rank	10	3.5	1.8	2.1	1.2
B8-12	20	19 (1)	18 (4)	19 (1)	19 (1)
BLS256	20	17 (4)	18 (3)	19 (2)	20 (1)
BLS279	20	16 (3)	15 (4)	17 (2)	20 (1)
BXOR1	20	17 (3)	16 (4)	18 (2)	19 (1)
CFBP2286	20	12 (3)	12 (3)	13 (1)	13 (1)
CFBP7331	20	9 (3)	10 (2)	9 (3)	12 (1)
CFBP7341	20	10 (2)	10 (2)	8 (4)	12 (1)
CFBP7342	20	13 (1)	11 (3)	12 (2)	11 (3)
L8	20	21 (3)	24 (2)	21 (3)	25 (1)
RS105	20	14 (2)	14 (2)	14 (2)	15 (1)
avg. rank	20	2.5	2.9	2.2	1.2
B8-12	50	23 (2)	25 (1)	22 (3)	22 (3)
BLS256	50	19 (4)	24 (1)	21 (3)	22 (2)
BLS279	50	20 (2)	20 (2)	19 (4)	22 (1)
BXOR1	50	20 (4)	21 (3)	22 (2)	23 (1)
CFBP2286	50	13 (4)	17 (1)	16 (2)	16 (2)
CFBP7331	50	12 (3)	12 (3)	13 (1)	13 (1)
CFBP7341	50	12 (2)	12 (2)	11 (4)	13 (1)
CFBP7342	50	14 (2)	14 (2)	15 (1)	13 (4)
L8	50	24 (4)	27 (1)	27 (1)	27 (1)
RS105	50	17 (4)	18 (1)	18 (1)	18 (1)
avg. rank	50	3.1	1.7	2.2	1.7
B8-12	TALEs AUC	901 (4)	971 (1)	912 (3)	924 (2)
BLS256	TALEs AUC	797 (4)	924 (2)	916 (3)	981 (1)
BLS279	TALEs AUC	757 (4)	800 (2)	775 (3)	949 (1)
BXOR1	TALEs AUC	776 (4)	818 (3)	888 (1)	887 (2)
CFBP2286	TALEs AUC	552 (4)	657 (3)	664 (1)	662 (2)
CFBP7331	TALEs AUC	432 (4)	517 (2)	469 (3)	562 (1)
CFBP7341	TALEs AUC	458 (3)	514 (2)	421 (4)	557 (1)
CFBP7342	TALEs AUC	589 (1)	535 (4)	553 (2)	540 (3)
L8	TALEs AUC	994 (4)	1163 (2)	1099 (3)	1186 (1)
RS105	TALEs AUC	638 (4)	732 (2)	704 (3)	752 (1)
avg. rank	TALEs AUC	3.6	2.3	2.6	1.5

Table R. Performance evaluation on the level of TALEs for ten *Xoo* strains when filtering for predictions of TALE boxes on the same strand as the downstream gene. For each strain and each approach, we list the number of TALEs with at least one predicted target gene that is also up-regulated in the infection for different cutoffs on the number of predictions per TALE, i.e., prediction ranks. For each threshold, we additionally report the average rank of each tool.

measure	Target Finder	TALgetter	Talvez	PrediTALE	Quade	TALgetter vs TALESF	Talvez vs Target Finder	Talvez vs TALgetter	PrediTALE vs Target Finder	PrediTALE vs TALgetter	PrediTALE vs Talvez
Genes R1	2.1	2.2	2.6	1.5							
Genes R10	3.5	2.1	2.7	1	***	++	++		+++	+++	+++
Genes R20	2.9	2.7	2.4	1.3	*				+++	+++	++
Genes R50	3.3	2.8	2.3	1.3	**		++	+	+++	+++	
Genes AUC	3.3	2.7	2.7	1.3	***	+	+		+++	+++	+++
TALEs R1	2.1	2.2	2.6	1.5							
TALEs R10	3.5	1.8	2.1	1.2	***	+++	+++		+++		+
TALEs R20	2.5	2.9	2.2	1.2	**				+++	+++	+++
TALEs R50	3.1	1.7	2.2	1.7	*	+++			++		
TALEs AUC	3.6	2.3	2.6	1.5	***	+++	+		+++	+	+++

Table S. Testing the significance of differences in prediction performance when filtering for predictions of TALE boxes on the same strand as the downstream gene. For each tool and each measure (TALEs/Genes; rank cutoff), we report the average rank per tool, the significance of the Quade test (*:< 0.05; **:< 0.01; ***:< 0.001), and the significance of the pairwise comparison in a post-hoc test. Here, '+' and '-' indicate that the first tool has gained a significantly better or worse performance than the second one, respectively. The number of symbols encodes the significance level in analogy to the Quade test.

Tool	Runtime (min:sec)
PrediTALE	24:34
Target Finder	09:21
Talvez	347:35
TALgetter	42:45

Table T. Runtime of all four approaches for genome-wide scans of the rice genome for target boxes of all 28 TALEs of strain *Xoc* BLS256.
