# Supplementary figures and images for: PrediTALE: A novel model learned from quantitative data allows for new perspectives on TALE targeting
Source: PLoS Comput Biol. 2019 Jul 11;15(7):e1007206. doi: 10.1371/journal.pcbi.1007206 (PMC6650089; doi:10.1371/journal.pcbi.1007206)

B8-12

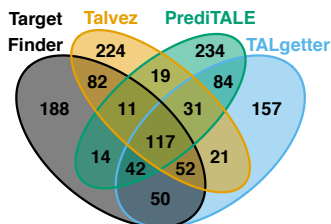

BLS256

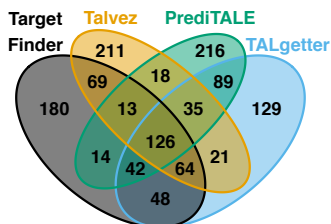

BLS279

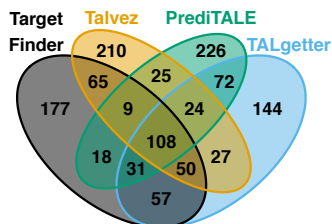

BXOR1

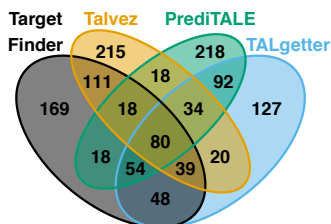

CFBP2286

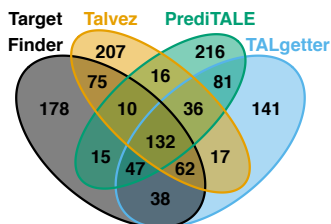

CFBP7331

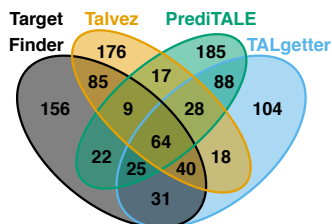

CFBP7341

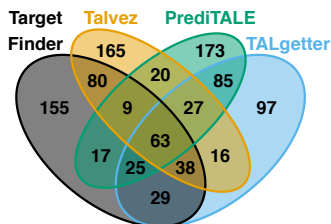

CFBP7342

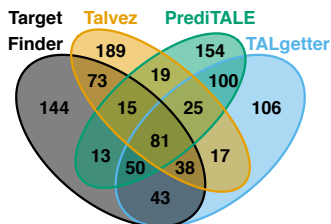

L8

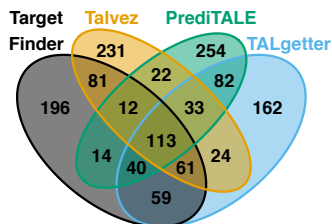

RS105

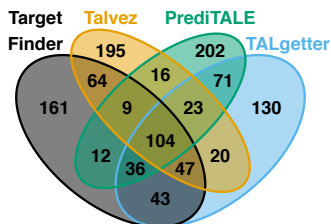

Supplement: S1 Fig — For each Xoc strain and each approach, we consider the set of target genes obtained as the union of the top 20 predictions per TALE. (PDF) [file pcbi.1007206.s010.pdf]

B8-12

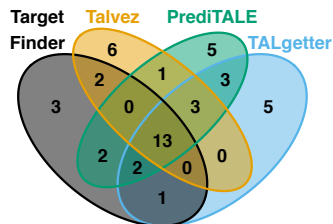

BLS256

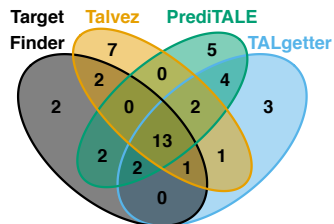

BLS279

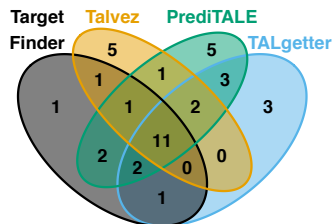

BXOR1

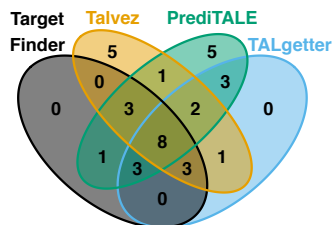

CFBP2286

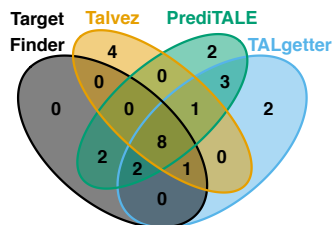

CFBP7331

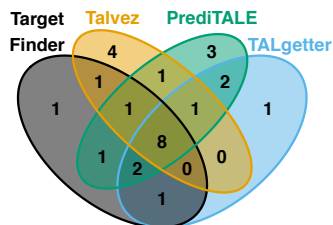

CFBP7341

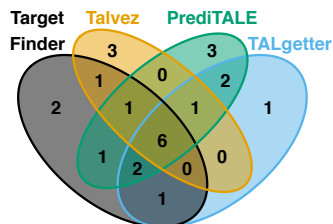

CFBP7342

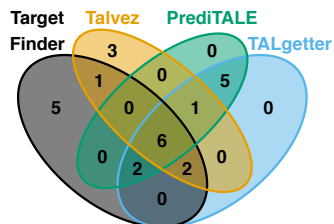

L8

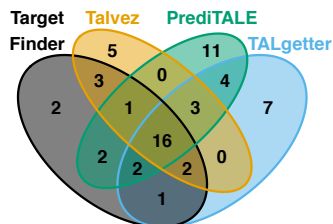

RS105

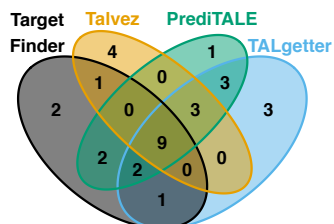

Supplement: S2 Fig — For each Xoc strain and each approach, we consider the set of target genes obtained as the union of the top 20 predictions per TALE. These sets are filtered by up-regulation of the corresponding genes according to RNA-seq data, and the resulting subsets are displayed. (PDF) [file pcbi.1007206.s011.pdf]

**ICMP 3125**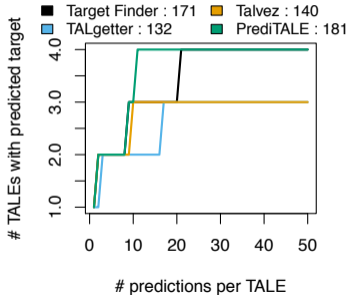**PXO142**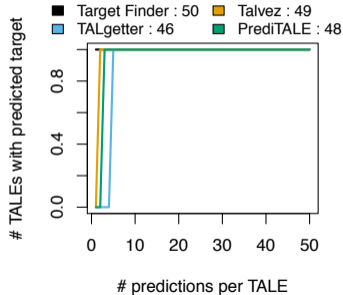**PXO83**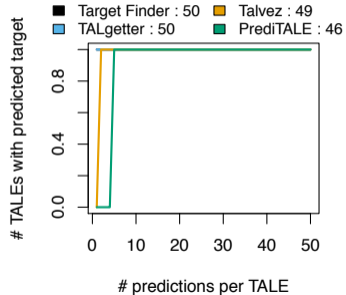

Supplement: S4 Fig — For each approach, we plot the number of TALEs with at least one predicted target gene that is also up-regulated in the infection (q-value < 0.01, log fold change > 2) against the number of predicted target sites per TALE. (PDF) [file pcbi.1007206.s013.pdf]

**ICMP 3125**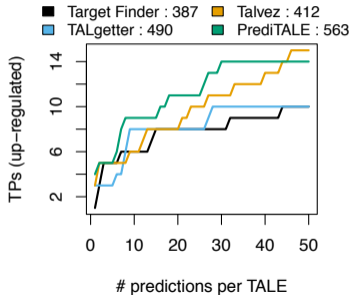**PXO142**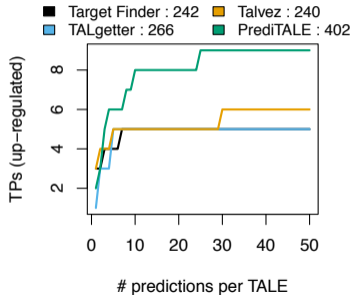**PXO83**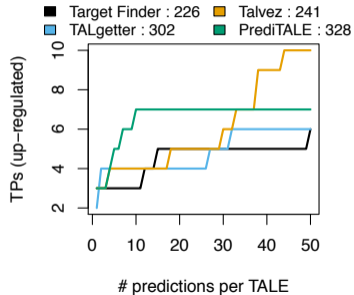

Supplement: S5 Fig — For each approach, we plot the number of predicted target genes that are also up-regulated in the infection (true positives, TPs) against the number of predicted target sites per TALE. (PDF) [file pcbi.1007206.s014.pdf]

**ICMP 3125**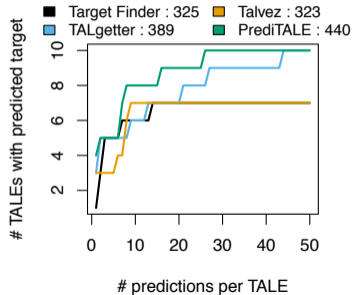**PXO142**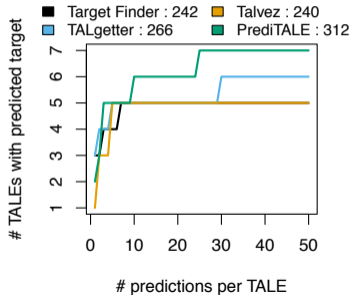**PXO83**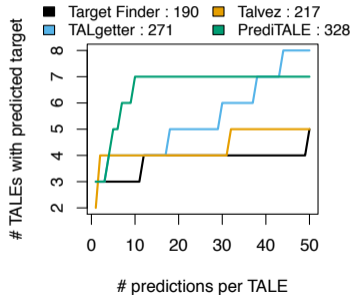

Supplement: S6 Fig — For each approach, we plot the number of TALEs with at least one predicted target gene that is also up-regulated in the infection against the number of predicted target sites per TALE. (PDF) [file pcbi.1007206.s015.pdf]

**B8-12**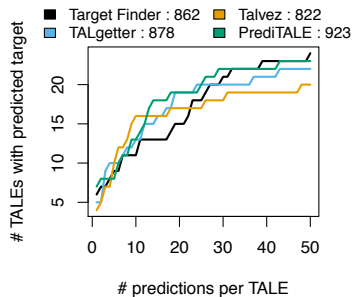**BLS256**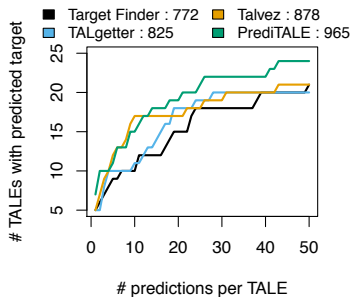**BLS279**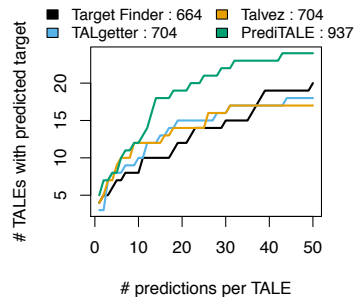**BXOR1**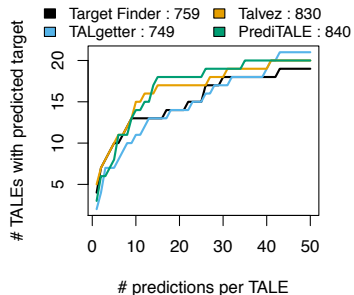**CFBP2286**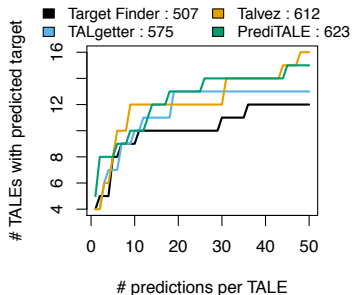**CFBP7331**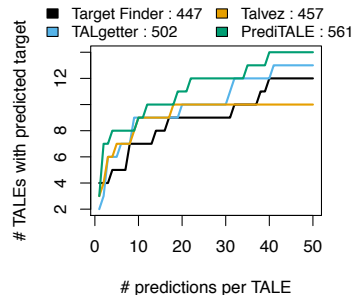**CFBP7341**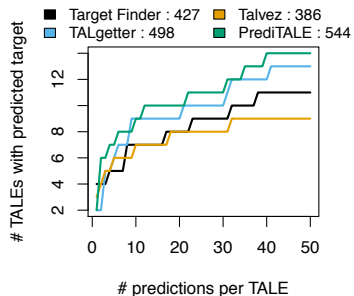**CFBP7342**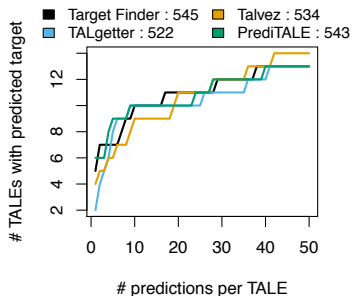**L8**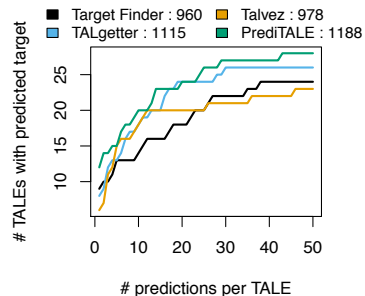**RS105**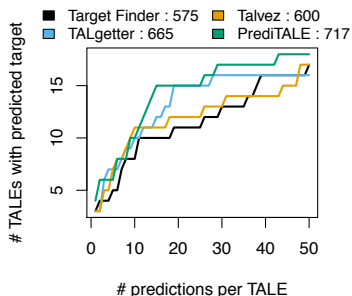

Supplement: S7 Fig — For each approach, we plot the number of TALEs with at least one predicted target gene that is also up-regulated in the infection against the number of predicted target sites per TALE. (PDF) [file pcbi.1007206.s016.pdf]

**B8-12**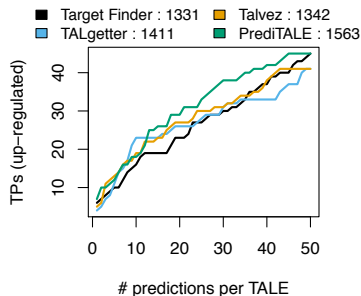**BLS256**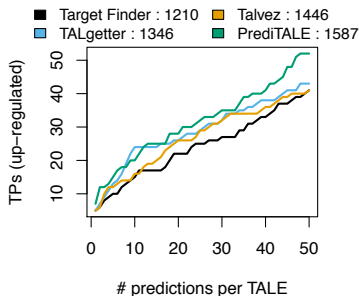**BLS279**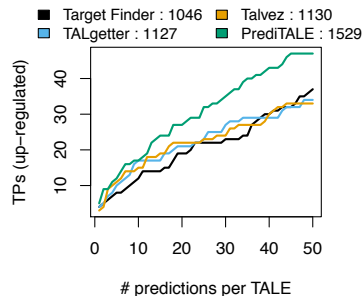**BXOR1**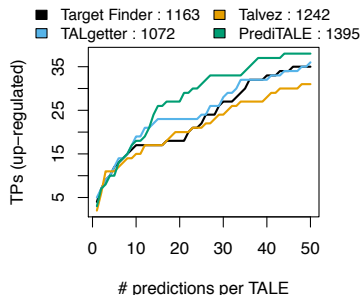**CFBP2286**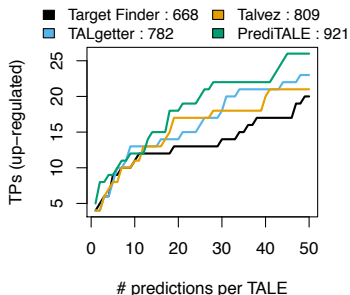**CFBP7331**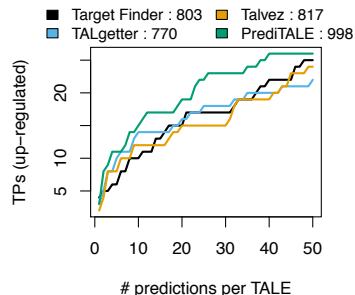**CFBP7341**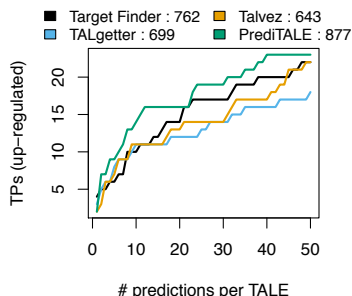**CFBP7342**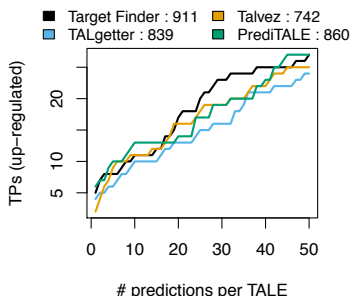**L8**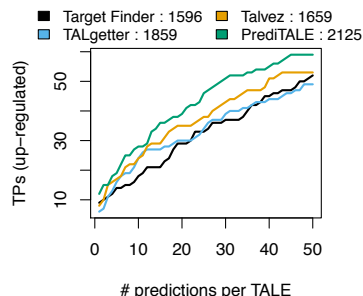**RS105**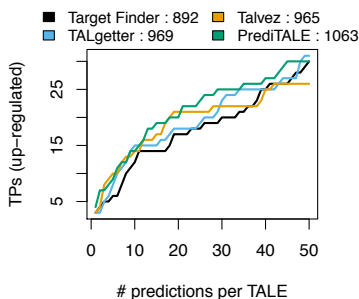

Supplement: S8 Fig — For each approach, we plot the number of predicted target genes that are also up-regulated in the infection (true positives, TPs; q-value < 0.05, log fold change > 2) against the number of predicted target sites per TALE. (PDF) [file pcbi.1007206.s017.pdf]

**B8-12**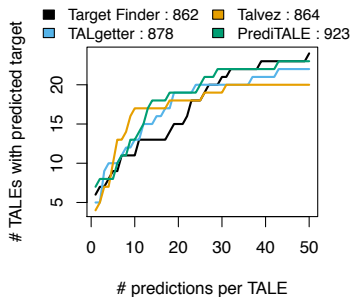**BLS256**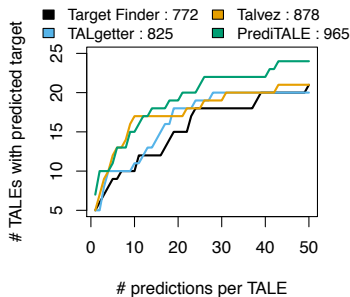**BLS279**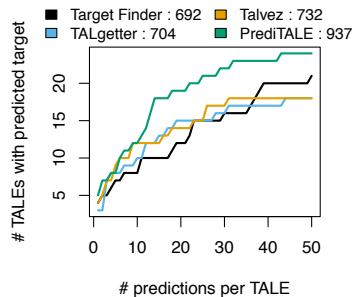**BXOR1**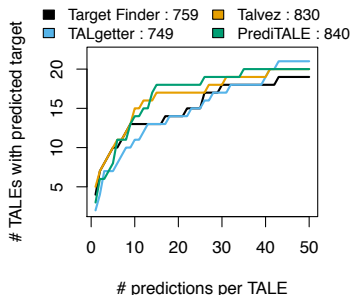**CFBP2286**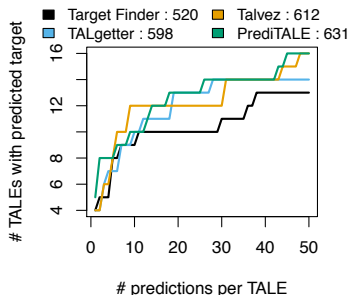**CFBP7331**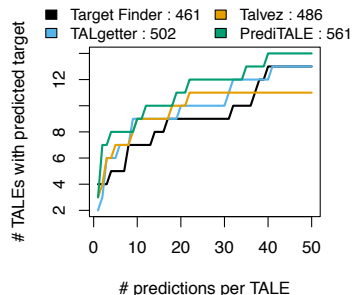**CFBP7341**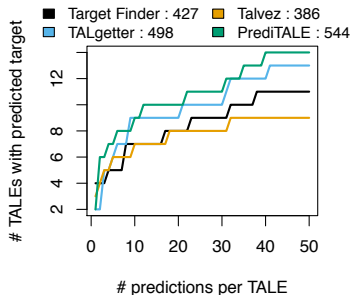**CFBP7342**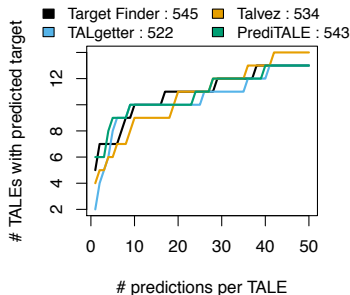**L8**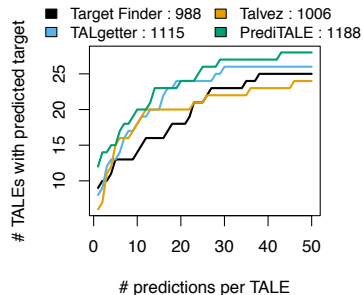**RS105**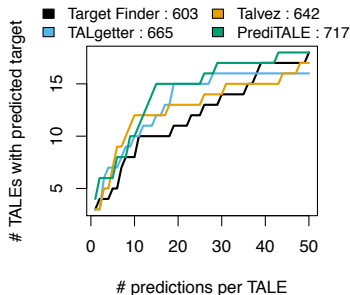

Supplement: S9 Fig — For each approach, we plot the number of TALEs with at least one predicted target gene that is also up-regulated in the infection (q-value < 0.05, log fold change > 2) against the number of predicted target sites per TALE. (PDF) [file pcbi.1007206.s018.pdf]

**B8-12**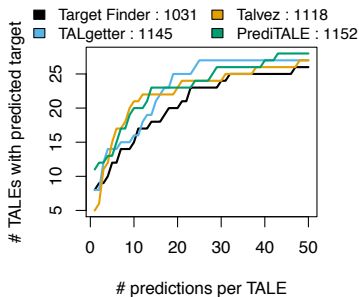**BLS256**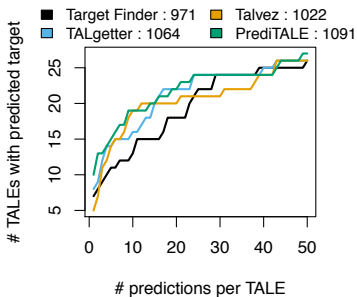**BLS279**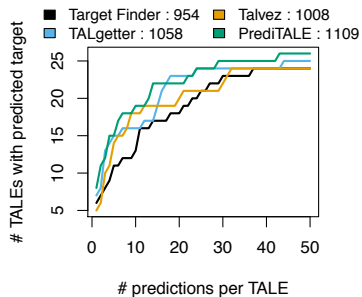**BXOR1**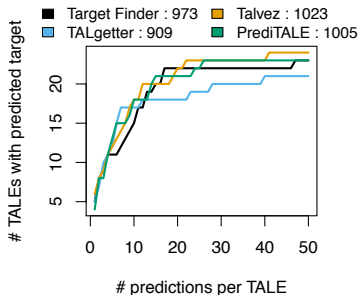**CFBP2286**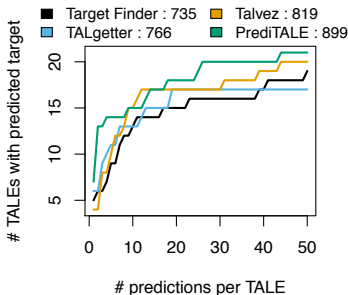**CFBP7331**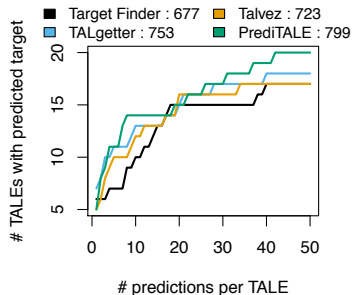**CFBP7341**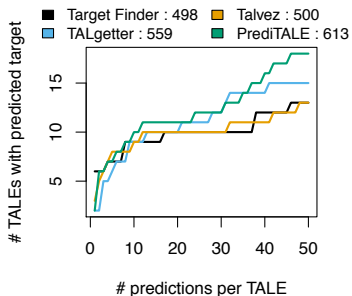**CFBP7342**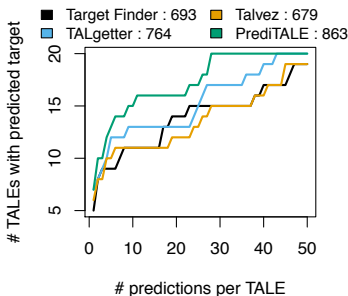**L8**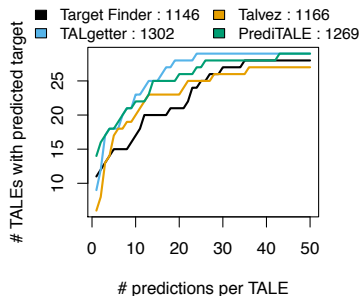**RS105**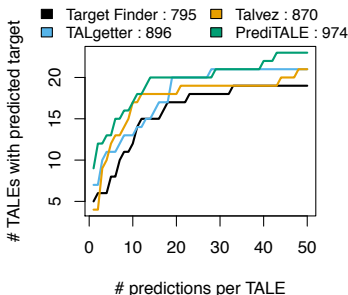

Supplement: S11 Fig — For each approach, we plot the number of TALEs with at least one predicted target gene that is also up-regulated in the infection (q-value < 0.01, log fold change > 1) against the number of predicted target sites per TALE. (PDF) [file pcbi.1007206.s020.pdf]

**B8-12**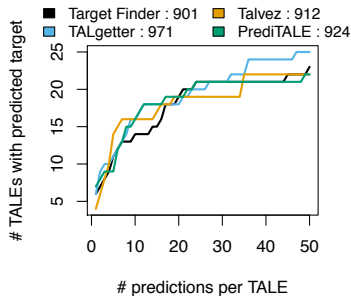**BLS256**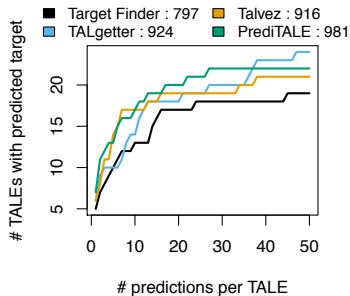**BLS279**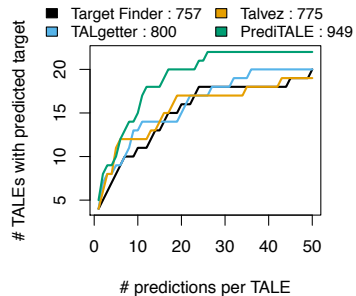**BXOR1**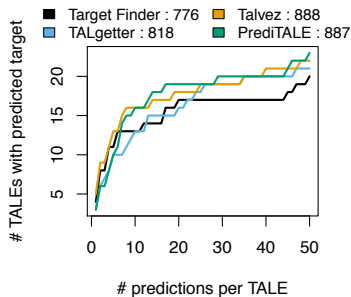**CFBP2286**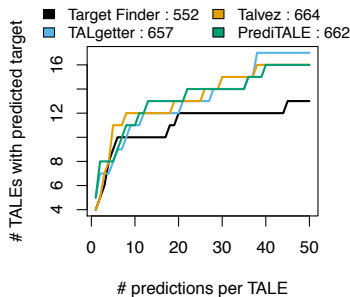**CFBP7331**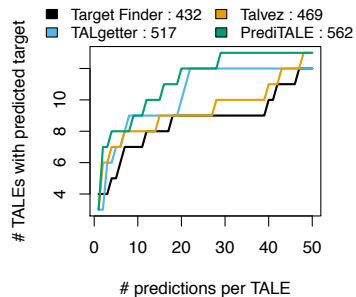**CFBP7341**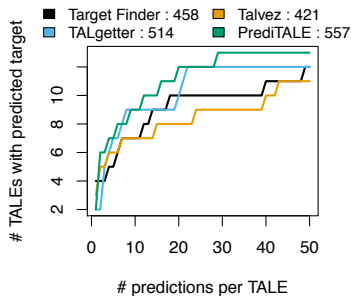**CFBP7342**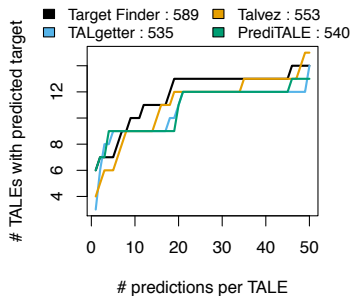**L8**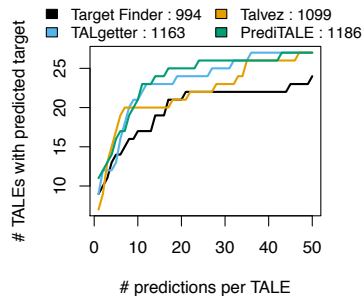**RS105**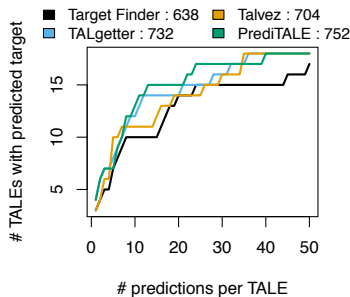

Supplement: S13 Fig — For each approach, we plot the number of TALEs with at least one predicted target gene that is also up-regulated in the infection against the number of predicted target sites per TALE. (PDF) [file pcbi.1007206.s022.pdf]

difference (TPs)

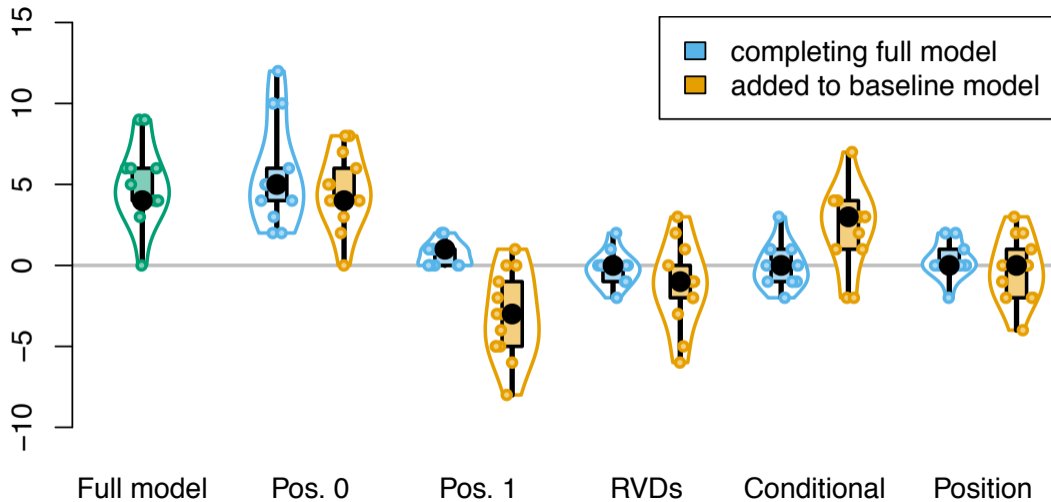

Supplement: S14 Fig — For each subset of features, we additionally compare the case where i) features are completing the full model and ii) features are added to the baseline model. We show violin plots of the number of true positive target gene predictions using at most 20 predictions per TALE including individual points for all Xoo and Xoc data sets. (PDF) [file pcbi.1007206.s023.pdf]

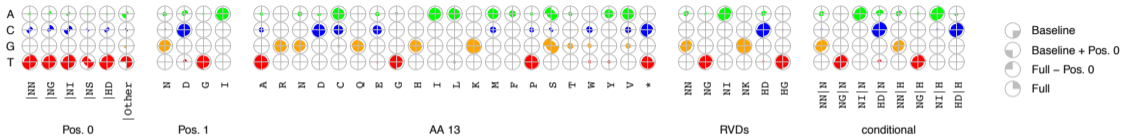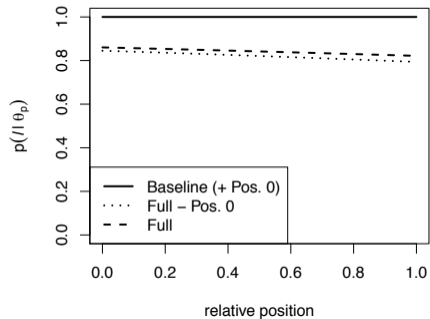

Supplement: S15 Fig — These are the baseline model using only specificities based on AA 13 of an RVD and independent parameters for position 0, a baseline model with conditional parameters for position 0 added, the full model except conditional parameters for position 0, and the full PrediTALE model. We find substantial differences between the specificity parameters of the four models, also in the parameters for specificities based on AA 13 of an RVD, although these are included into all four models. By contrast, we do not find a major difference between the position distributions learned for the full model and the full model except conditional parameters for position 0. (PDF) [file pcbi.1007206.s024.pdf]

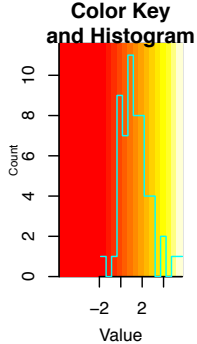

|          |        |        |            |
|----------|--------|--------|------------|
| 1.894    | 0.887  | 0.737  | Os01g40290 |
| 1.079    | 0.007  | -0.044 | Os01g73890 |
| 3.815    | 1.867  | 2.739  | Os02g06670 |
| -0.009   | 5.163  | 0.162  | Os02g49350 |
| 1.295    | 0.820  | 1.181  | Os03g03034 |
| -0.791   | 2.530  | 2.181  | Os03g09150 |
| 2.734    | 1.368  | 1.914  | Os03g51760 |
| 2.221    | 1.387  | 1.621  | Os04g05050 |
| -0.164   | 0.065  | 1.700  | Os04g19960 |
| 5.762    | -0.163 | 0.311  | Os04g43730 |
| 1.704    | 0.169  | 0.043  | Os05g45070 |
| 1.591    | 1.183  | 0.506  | Os06g03710 |
| 1.902    | 0.833  | 0.690  | Os06g29790 |
| 0.687    | 0.824  | 1.398  | Os07g06970 |
| 0.746    | 0.042  | 0.039  | Os09g07460 |
| 2.819    | 2.272  | 2.825  | Os09g29820 |
| 0.918    | 0.224  | 0.265  | Os10g28240 |
| 1.695    | 1.087  | 0.477  | Os11g26790 |
| -1.882   | 2.514  | 3.819  | Os11g31190 |
| ICMP3125 | PXO142 | PXO83  |            |

Supplement: S17 Fig — (PDF) [file pcbi.1007206.s026.pdf]

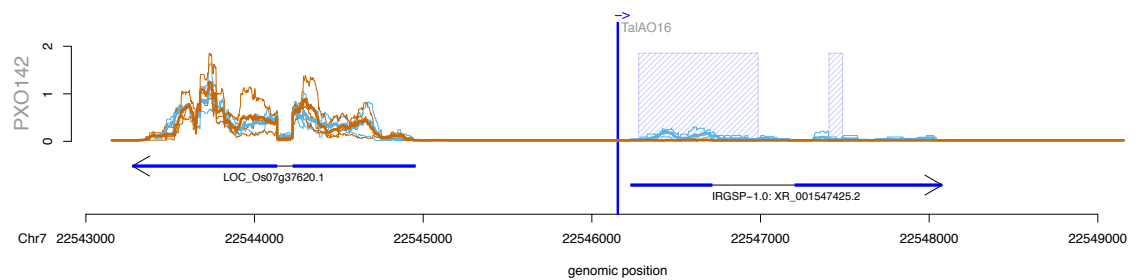

Supplement: S18 Fig — RNA-seq coverage after inoculation (blue line) is compared with mock control (brown line). In addition, we show the average of individual replicates of control and treatment are summarized as thick lines. The blue shaded boxes mark the differentially expressed regions. The arrows under the profiles reflect the MSU7 annotation within the genomic region. The genomic position of the TALE target box is marked by a vertical blue line. (PDF) [file pcbi.1007206.s027.pdf]
