# Supplementary material for: PrediTALE: A novel model learned from quantitative data allows for new perspectives on TALE targeting
Source: PLoS Comput Biol. 2019 Jul 11;15(7):e1007206. doi: 10.1371/journal.pcbi.1007206 (PMC6650089; doi:10.1371/journal.pcbi.1007206)
Supplement: S3 Fig — For each approach, we plot the number of predicted target genes that are also up-regulated in the infection (true positives, TPs; q-value < 0.01, log fold change > 2) against the number of predicted target sites per TALE. (PDF) [file pcbi.1007206.s012.pdf]

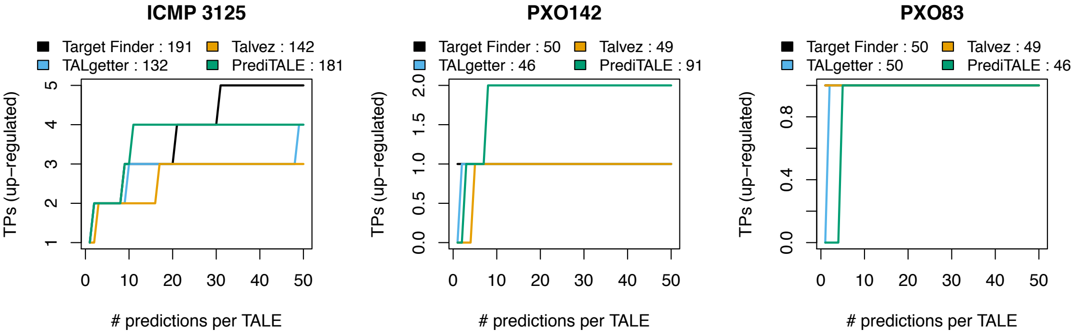
ICMP 3125
Target Finder : 191
Talvez : 142
TALgetter : 132
PrediTALE : 181
TPs (up−regulated)
predictions per TALE
PXO142
Target Finder : 50
Talvez : 49
TALgetter : 46
PrediTALE : 91
TPs (up−regulated)
predictions per TALE
PXO83
Target Finder : 50
Talvez : 49
TALgetter : 50
PrediTALE : 46
TPs (up−regulated)
predictions per TALE
