# Supplementary material for: PrediTALE: A novel model learned from quantitative data allows for new perspectives on TALE targeting
Source: PLoS Comput Biol. 2019 Jul 11;15(7):e1007206. doi: 10.1371/journal.pcbi.1007206 (PMC6650089; doi:10.1371/journal.pcbi.1007206)
Supplement: S10 Fig — For each approach, we plot the number of predicted target genes that are also up-regulated in the infection (true positives, TPs; q-value < 0.01, log fold change > 1) against the number of predicted target sites per TALE. (PDF) [file pcbi.1007206.s019.pdf]

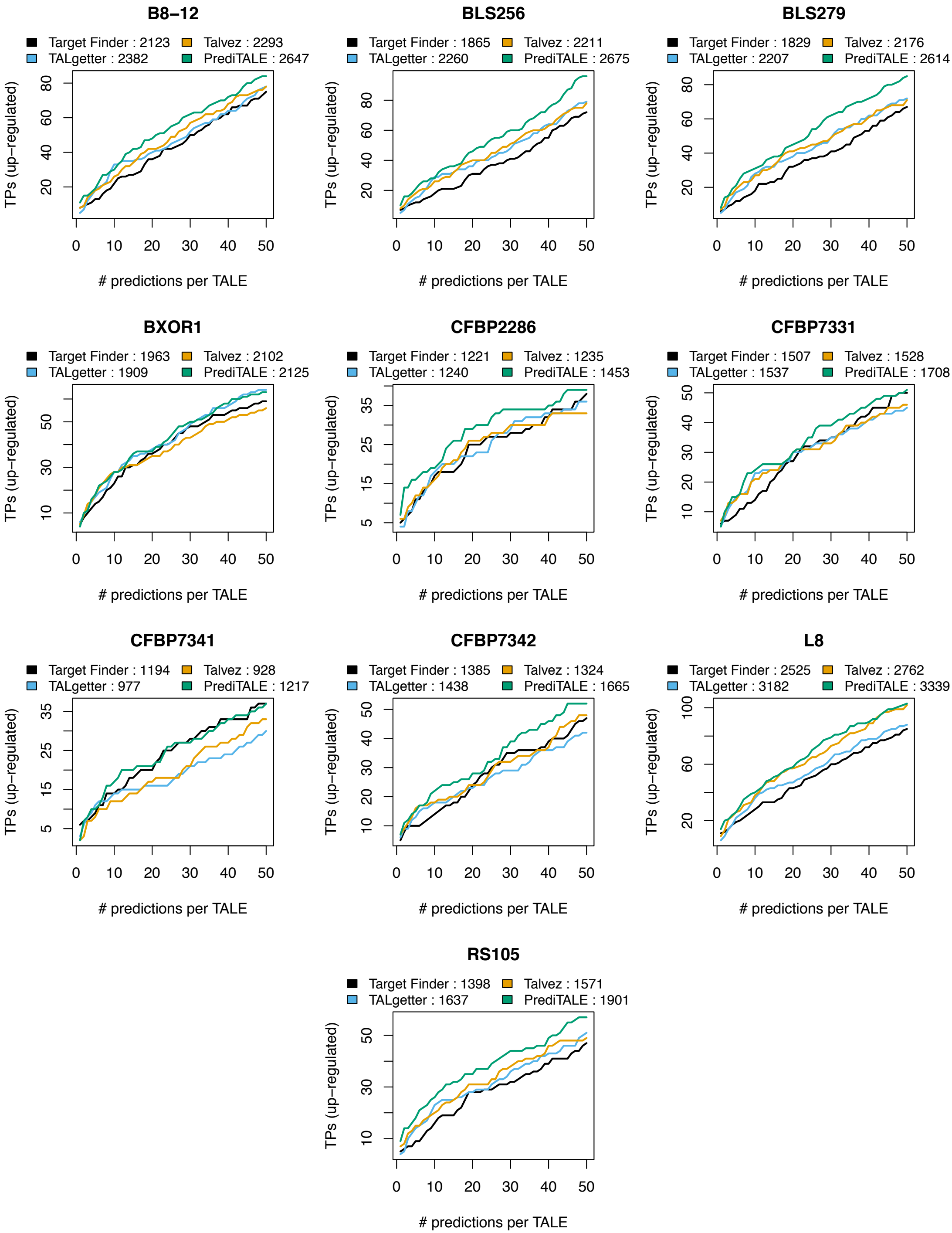
B8−12
Target Finder : 2123
Talvez : 2293
TALgetter : 2382
PrediTALE : 2647
BLS256
Target Finder : 1865
Talvez : 2211
TALgetter : 2260
PrediTALE : 2675
BLS279
Target Finder : 1829
Talvez : 2176
TALgetter : 2207
PrediTALE : 2614
BXOR1
Target Finder : 1963
Talvez : 2102
TALgetter : 1909
PrediTALE : 2125
CFBP2286
Target Finder : 1221
Talvez : 1235
TALgetter : 1240
PrediTALE : 1453
CFBP7331
Target Finder : 1507
Talvez : 1528
TALgetter : 1537
PrediTALE : 1708
CFBP7341
Target Finder : 1194
Talvez : 928
TALgetter : 977
PrediTALE : 1217
CFBP7342
Target Finder : 1385
Talvez : 1324
TALgetter : 1438
PrediTALE : 1665
L8
Target Finder : 2525
Talvez : 2762
TALgetter : 3182
PrediTALE : 3339
RS105
Target Finder : 1398
Talvez : 1571
TALgetter : 1637
PrediTALE : 1901
TPs (up−regulated)
predictions per TALE
