# Supplementary material for: PrediTALE: A novel model learned from quantitative data allows for new perspectives on TALE targeting
Source: PLoS Comput Biol. 2019 Jul 11;15(7):e1007206. doi: 10.1371/journal.pcbi.1007206 (PMC6650089; doi:10.1371/journal.pcbi.1007206)
Supplement: S12 Fig — For each approach, we plot the number of predicted target genes that are also up-regulated in the infection (true positives, TPs) against the number of predicted target sites per TALE. (PDF) [file pcbi.1007206.s021.pdf]

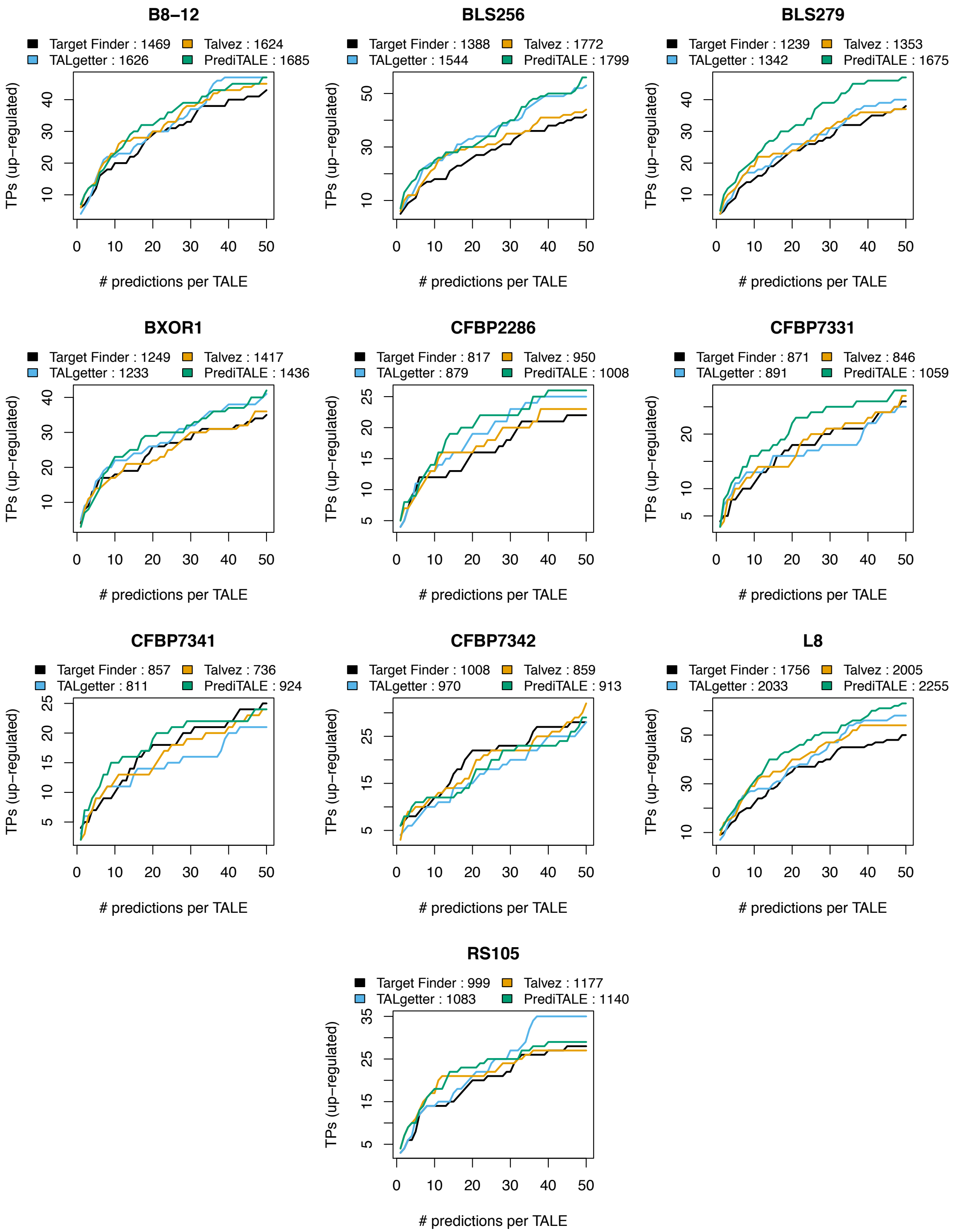
B8–12
Target Finder : 1469
Talvez : 1624
TALgetter : 1626
PrediTALE : 1685
BLS256
Target Finder : 1388
Talvez : 1772
TALgetter : 1544
PrediTALE : 1799
BLS279
Target Finder : 1239
Talvez : 1353
TALgetter : 1342
PrediTALE : 1675
BXOR1
Target Finder : 1249
Talvez : 1417
TALgetter : 1233
PrediTALE : 1436
CFBP2286
Target Finder : 817
Talvez : 950
TALgetter : 879
PrediTALE : 1008
CFBP7331
Target Finder : 871
Talvez : 846
TALgetter : 891
PrediTALE : 1059
CFBP7341
Target Finder : 857
Talvez : 736
TALgetter : 811
PrediTALE : 924
CFBP7342
Target Finder : 1008
Talvez : 859
TALgetter : 970
PrediTALE : 913
L8
Target Finder : 1756
Talvez : 2005
TALgetter : 2033
PrediTALE : 2255
RS105
Target Finder : 999
Talvez : 1177
TALgetter : 1083
PrediTALE : 1140
TPs (up−regulated)
predictions per TALE
