# Supplementary material for: PrediTALE: A novel model learned from quantitative data allows for new perspectives on TALE targeting
Source: PLoS Comput Biol. 2019 Jul 11;15(7):e1007206. doi: 10.1371/journal.pcbi.1007206 (PMC6650089; doi:10.1371/journal.pcbi.1007206)
Supplement: S16 Fig — (PDF) [file pcbi.1007206.s025.pdf]

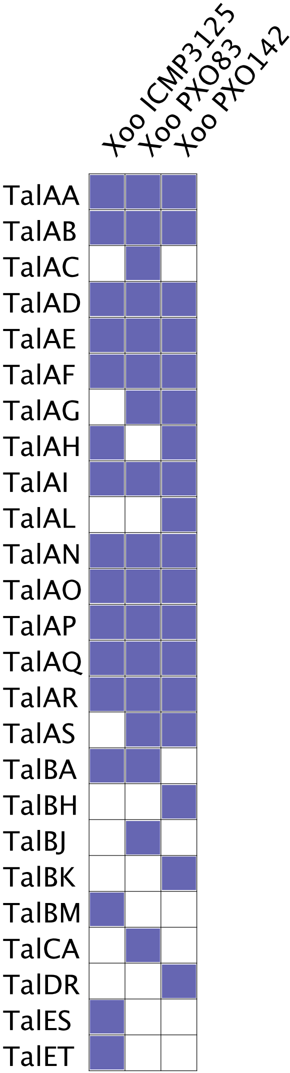
Xoo ICMP3125
Xoo PXO83
Xoo PXO142
TalAA
TalAB
TalAC
TalAD
TalAE
TalAF
TalAG
TalAH
TalAI
TalAL
TalAN
TalAO
TalAP
TalAQ
TalAR
TalAS
TalBA
TalBH
TalBJ
TalBK
TalBM
TalCA
TalDR
TalES
TalET
